# Towards Understanding Factors Affecting Arsenic, Chromium, and Vanadium Mobility in the Subsurface

**DOI:** 10.3390/w14223687

**Published:** 2022-11-15

**Authors:** Hannah R. Peel, Fatai O. Balogun, Christopher A. Bowers, Cass T. Miller, Chelsea S. Obeidy, Matthew L. Polizzotto, Sadeya U. Tashnia, David S. Vinson, Owen W. Duckworth

**Affiliations:** 1 Department of Crop and Soil Sciences, North Carolina State University, Raleigh, NC 27695, USA; 2 Department of Earth Sciences, University of Oregon, Eugene, OR 97403, USA; 3 Department of Environmental Sciences and Engineering, University of North Carolina, Chapel Hill, NC 27599, USA; 4 Department of Geography and Earth Sciences, University of North Carolina at Charlotte, Charlotte, NC 28223, USA

**Keywords:** geogenic contaminants, hydrogeochemistry, speciation, mechanistic modeling

## Abstract

Arsenic (As), chromium (Cr), and vanadium (V) are naturally occurring, redox-active elements that can become human health hazards when they are released from aquifer substrates into groundwater that may be used as domestic or irrigation source. As such, there is a need to develop incisive conceptual and quantitative models of the geochemistry and transport of potentially hazardous elements to assess risk and facilitate interventions. However, understanding the complexity and heterogeneous subsurface environment requires knowledge of solid-phase minerals, hydrologic movement, aerobic and anaerobic environments, microbial interactions, and complicated chemical kinetics. Here, we examine the relevant geochemical and hydrological information about the release and transport of potentially hazardous geogenic contaminants, specifically As, Cr, and V, as well as the potential challenges in developing a robust understanding of their behavior in the subsurface. We explore the development of geochemical models, illustrate how they can be utilized, and describe the gaps in knowledge that exist in translating subsurface conditions into numerical models, as well as provide an outlook on future research needs and developments.

## 1. Introduction

Potentially toxic metal(loid)s in groundwater pose a significant threat to human health. A number of potentially toxic elements can be released from geomaterials into groundwater, including arsenic (As), chromium (Cr), vanadium (V), selenium (Se), fluorine (F), molybdenum (Mo), manganese (Mn), and boron (B) [1–10]. The most well-known geogenic contamination of groundwater occurs in Southern Asia, where up to 75 million people are estimated to be at risk of increased disease burden due to drinking-wells drawing from a shallow aquifer containing high As concentrations, a situation that has been described as “the largest mass poisoning in history” [11]. Worldwide, more than 150 million people drink water from wells with arsenic (As) concentrations greater than the USEPA and WHO maximum level of 10 μg/L [12,13]. Arsenic is a well-documented carcinogen, affecting the skin, liver, kidneys, lungs, and bladder. Chronic exposure is frequently associated with skin lesions, cardiovascular and respiratory disorders, reproductive failures, insulin resistance, and neurotoxicity [12–14]. Although As is often used as an example of an element that exhibits chronic toxicity and is globally widespread, related oxyanion-forming trace elements (e.g., Cr and V [15–18], which, along with As, we focus on in this work), are also naturally elevated in specific regions across the globe. For example, Cr (as hexavalent Cr) is a dangerous carcinogen, teratogen, and mutagen that has been found to occur naturally in groundwater systems, including in the continental US [7,19]; although the toxicology of V is less understood [20], this element can be very mobile under naturally occurring conditions [21] and is emerging as an environmental concern [22]. Despite the catastrophic size of the problem, global sustainable development goals do not consider As [23], or other geogenic contaminants, as a factor in supplying drinking water to communities worldwide.

Drinking water is a primary route of exposure for As, often from groundwater, which more than 2 billion people worldwide use as their primary domestic water source [24,25]. This dependence, along with the fact that >99% of accessible freshwater resides in groundwater [26], makes understanding, predicting, and managing of groundwater of foremost importance. However, subsurface processes are notoriously poorly understood, and controlled by coupled hydrogeochemical and biogeochemical processes, often involving kinetic or transport limitations, microbial processes, and interfacial reactions. In general, the solubilization of the potentially toxic contaminants in aquifers, be they geogenic or anthropogenic in origin, are thought to be controlled by phase transformations that are mediated thought redox, precipitation, dissolution, and (de)sorption reactions, all influenced by pH, ionic strength, and the presence of other constituents [3,5,9,16,18]. Beyond chemical reactions, physical considerations, such as lithologic heterogeneity or hydrologic complexity, limit our understanding of subsurface processes impacting contaminant behavior.

These complex physicochemical factors limit our ability to produce comprehensive and robust conceptual models, and hence reliable quantitative models, that represent our understanding of geogenic contamination in the subsurface and guide management to protect human health. The goal of this work is to review the current state of knowledge, identify key challenges, and provide an outlook for resolving issues associated with assessing and modeling inorganic geogenic contaminants, including As, Cr, and V, in subsurface environments that are sources of drinking water. This article aims to: (1) consider the groundwater hydrology and its effect on transport and distribution; (2) discuss geogenic sources of these contaminants; (3) investigate geochemical mechanisms affecting contrasting contaminant behavior of metal(loid)s; (4) describe methods for determining the concentration and speciation of metal(loid)s in water and geomaterials; (5) discuss approaches for deriving mechanistic models of contaminant transport and fate; and (6) conclude with a reflection on our needs and an outlook for future activities. Understanding and creating conceptual models of the release, retention, and transport of geogenic contaminants in groundwater systems, will ultimately enable the creation of quantitative models that can be used to predict future states and guide efforts to remediate contaminated groundwater to protect human health.

## 2. Framing Subsurface Hydrogeochemical Processes

### 2.1. Groundwater Flow and Its Relationship to Chemical Evolution

**Figure 1.**
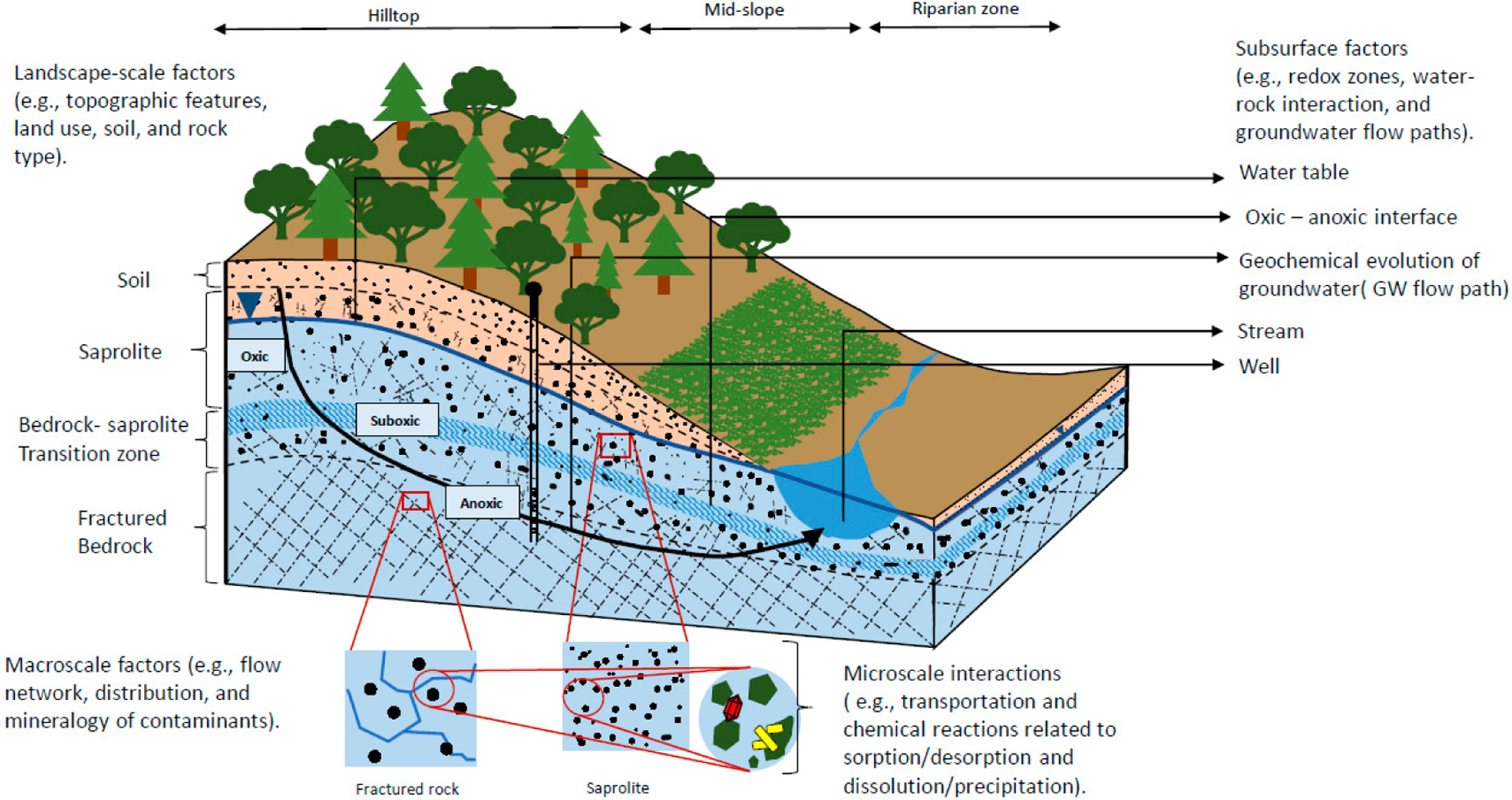
Conceptual model showing geochemical evolution of groundwater in the saturated and unsaturated zones, including the hydrogeochemical factors that influence the contaminant chemistry at multiple scales. Dashed lines indicate lithologic boundaries (modified from [27–29]).

To understand the chemical release and mobility of contaminants in aquifers, it is first necessary to develop conceptual models of groundwater flow and how physical factors such as flow velocity, mixing, and topography influence its geochemical evolution (Figure 1). Different scenarios are needed to understand how the master variables Eh and pH change over space and time, as these parameters often exert dominant control over the geochemical processes that result in contaminant solubilization or sequestration in the subsurface.

Groundwater in confined and unconfined aquifers occupies distinct environments for oxic-anoxic relationships and trace element behavior. In unconfined aquifers, the water table is an interface between saturated and unsaturated zones where inputs of fresh oxic water and soil gases can occur along the flow path. Therefore, as an interface between saturated and unsaturated conditions, the water table is also an interface that modulates the redox state and pH of shallow groundwater via open system inputs of oxygen (O_2_) and carbon dioxide (CO_2_). However, anoxic groundwater is not unusual in unconfined aquifers where electron donors such as organic carbon can scavenge O_2_ [28–30]. In contrast, the upper interface of a confined aquifer is defined by a relatively impermeable formation, rather than the water table. Therefore, age and redox conditions in confined aquifers are associated with distance along the flow path, with groundwater becoming more reducing away from the recharge zone as it encounters electron donors.

**Figure 2.**
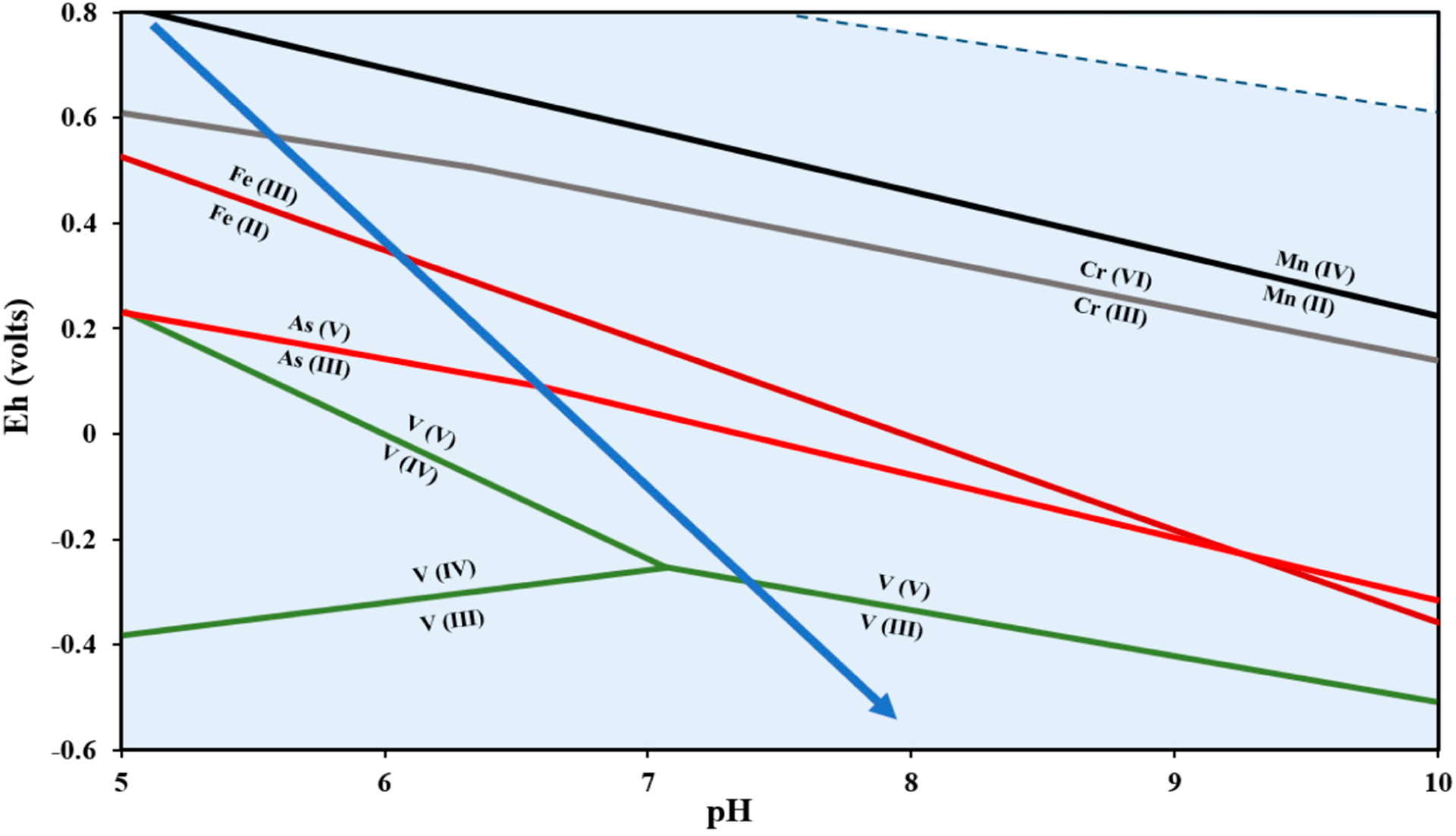
Eh-pH diagram for redox-sensitive elements at 25 °C showing he relative positions of redox boundaries for Mn, Fe, Cr, V, and As. The blue arrow indicates the generalized chemical evolution of groundwater chemistry. Total [Cr], [V], and [As] = 10^−7^ M each, total [Mn] and [Fe] = 10^−6^ M each (thermodynamic constants from [33–36]).

Groundwater chemistry evolves in terms of pH, redox state, and major ion concentrations. From the water table, recharging waters are predominantly oxic, with acidic pH and low alkalinity [31,32] but become more alkaline and reducing after encountering subsurface materials (including organic matter or reduced mineral phases), which in turn impact the speciation and movement of As, Cr and V (Figure 2). However, specific flow paths may be complex, incorporating mixtures of groundwater from different sources.

The topography of a landscape impacts groundwater flow patterns and may also have some association with groundwater redox gradients. Where confining units are absent, groundwater recharge augments flow along a flowpath. Accordingly, waters at mid- to high elevations on a landscape are dominated by recharge water whereas waters at mid- to low elevations on a landscape represent a mix between recharge and existing flowpath. Increasing distance blow the water table can promote development of more suboxic to anoxic and more alkaline conditions, as reductants and other reactive minerals are encountered (Figure 1). In discharge zones characterized by low topography in unconfined aquifers, groundwater flow can be significantly upward-directed, bringing more evolved, anoxic waters closer to the land surface [29].

Oxic and anoxic groundwater can be separated by centimeters to tens of meters vertically—capable of mixing across the scale of a well screen—and meters to hundreds of meters along a flow path (although key microscale redox variation may occur at the pore scale [37,38]). Groundwater evolves toward redox interfaces both vertically where groundwater flows predominantly downward (e.g., hilltop areas) and laterally where groundwater predominantly flows toward local stream valleys. These trends suggest the presence of the oxic-anoxic interface as a third critical interface along with the regolith-bedrock interface and the water table, which together contribute to landscape-scale heterogeneity of groundwater chemical conditions [15,39]. This pattern also suggests that depth and topographic position (hilltop vs. valley) could be master variables for approximating groundwater chemical evolution in an unconfined aquifer [40]. In addition to flow direction, there is nominal evidence that groundwater flow velocity can impact the concentration of redox constituents in groundwater; for example, a study in a shallow aquifer in Greece found that slower groundwater flow was correlated with higher concentrations of Cr(IV) due to a longer period of water-rock interaction [41].

Although the geology and hydrology of an area sets up the transition between oxic surficial inputs and electron donors hosted in the aquifer and forms the framework for groundwater redox evolution, anthropogenic inputs can alter groundwater redox evolution. Mixing can introduce oxic waters into a reduced environment, or reduced waters into an oxic environment, altering the overall chemistry. In pumped aquifers, mixing of disparate redox states can occur across long well screens [28]. Additional sources of electron acceptors (e.g., O_2_ or nitrate (NO_3_^−^)) or donors can result from surficial inputs by human land use activities. These can include organic materials, fertilizers, inorganic contaminants, petroleum, etc., and each will impart their own effect on the redox environment by their reactions and interactions with aqueous substrates, microorganisms, and existing groundwater conditions [42–45].

### 2.2. Limitations and Challenges

Recent studies on occurrence and health effects suggest that a large number of wells and aquifers are of concern for As or another naturally occurring element [12]. This situation is suited to probabalistic modeling approaches (e.g., probability of exceeding a water quality standard [15]), but mechanistic interpretation and modeling that could inform prediction at the scale of a water well are significantly more challenging. Several gaps in knowledge and practice are apparent for translating element occurrence to improved understanding of expected trace element behavior in well water. First, solid-phase geochemistry and mineralogy may only be known from a few sites in a region that contains many wells. In practice, the solid-phase level of an element of interest might be represented only approximately, for example by a map-scale geologic unit in which a well is located. Second, vertical heterogeneity can be considerable at the scale of a well’s open interval, which would create difficulty for translating element mobility concepts derived at the microscale/pore scale to the redox evolution that occurs along the scale of a realistic flowpath. Third, groundwater in a shallow unconfined aquifer represents a mix of multiple water sources of different residence times and flow lengths. This problem of along-flow and vertical heterogeneity is apparent in studies showing spatial variability of As, Cr, or other elements across the landscape, as discussed above. Heterogeneity’s effects on water chemistry might be especially significant when analyzing a pumped water sample across a large open interval, which can induce mixing of different water sources and chemistries. Groundwater flowpaths and mixing relationships can be inferred broadly from regional and topographic generalizations [40], but the flow path or mixing relationships of a pumped water sample may not be known for wells outside of well-characterized research sites. Collectively, these factors imply that the multiple geochemical parameters within a water sample (e.g., redox-sensitive solutes, trace element concentrations, speciation) are encoded with richer geochemical process information than the other more general attributes in field-based studies, such as well depth, landscape position, map-scale geological unit, or solid-phase composition from limited solids analysis. Therefore, there is a gap between understanding the cumulative net geochemical processes that resulted in a water sample’s observed chemistry, and predicting where in an aquifer, and at what rate these effects are imparted on groundwater.

## 3. Mineralogical Sources of Geogenic Contamination

Anthropogenic activities, such as electroplating, metallurgy, chemical production, energy production, pest control, and waste disposal, are common activities that result in contamination by metal(loid)s [46]. However, in the last two decades, there has been a growing recognition of the widespread importance of geogenic sources of these contaminants in groundwater [8]. This evolution in thinking is critical; a relatively low concentration of an element in geomaterial has the potential to contaminate groundwater for thousands of years under normal conditions [9]. However, the relative mobility of these elements is strongly controlled by what phases harbor contaminants, as well as the water chemistry of aquifer. Mineralogical information has not historically been considered by regulators, who are primarily concerned with the composition of the groundwater, and the presence of aqueous-phase hazards [47]. Generally, aquifer solids consist of multiple minerals, each containing different levels of As, Cr, or V, and having a wide range of weathering rates. We thus discuss the mineralogical sources of geogenic As, Cr, and V as a prelude to reviewing biogeochemical processes associated with their solubilization and transport. It should be noted that the factors that solubilize the elements may also redistribute them to more dilute, labile solid phase associations, such as adsorbed to clay mineral or (oxhydr)oxide phases, resulting in potentially new, potentially diffuse sources of contamination [48].

### 3.1. Arsenic

As is a ubiquitous element in the Earth’s crust and the 20th most abundant; concentrations in igneous and sedimentary rocks are typically 2–3 mg/kg, but concentrations can be higher in clayey sediments, phosphorites, and reduced marine sediments [49]. Geogenic As is often hosted mainly by mineral phases in metavolcanics, mudstones and their alteration products. There are over 500 As bearing minerals, including sulfides, oxides, arsenates and arsenites [50]. Because As can be coprecipitated with iron (Fe) (oxyhydr)oxide and sulfides in sedimentary rocks, and sedimentary Fe ores and Mn nodules are considered As-rich materials [49]. The most common and most studied are arsenopyrite (FeAsS) and As-bearing pyrite (FeS_2_), where As can substitute for S in the pyrite crystal structure. Globally, As rich ore deposits are found in China and Morocco, as well as the US, Russia, Japan, Iran and Belgium [51,52].

Arsenic contamination to groundwater derives not just from mineralized deposits, but also from hydrothermal activity (as could be found in Yellowstone, USA) and from aquifer sediments associated with silicic volcanic aquifers, and tuffaceous sediments and in sedimentary basins derived from these volcanic rock types [4,52–54]. The conditions under which As in released from minerals into a groundwater system are varied based on the source material. Thus, contamination that results from reducing groundwater environments has occurred in Northern China, Taiwan, Vietnam, Hungary, Romania, Slovakia, Bangladesh, southwestern US, and India. Conversely, in Mexico, Chile, Argentina, and the southwestern US, arid and oxidizing environments trigger the release of As to groundwater, highlighting the need to understand the biogeochemical processes associated with contamination in an aquifer [51,52].

### 3.2. Chromium

Cr, the 21st most abundant element in the Earth’s crust [55], occurs at an average concentration of around 100 mg/kg [41], typically as oxides and silicates [7,19]. In rock forming minerals, Cr pyroxenes, amphiboles, diopside, serpentine and chlorite are the chief hosts of Cr, where up to 1.5% Cr_2_O_3_ have been reported [56,57]. In Cr-bearing spinels, commonly chromite (FeCr_2_O_4_), magnesiochromite (MgCr_2_O_4_), and magnetite (Fe_3_O_4_), the percentage Cr could be up to 35%, often presenting as Cr_2_O_3_ [41,56]. Alteration of primary silicate minerals during metamorphic events may produce minerals such as fuchsite, uvarovite, Cr-mica and tawmawite, where Cr substitutes for Al in the octahedral site through isomorphism [19]. Globally, chromite is the most common deposit of Cr, found circum-pacific and the Mediterranian [7] as well in South Africa, Khazahstan, Zimbabwe, and India [55]. It is generally associated with ultramafic rocks and their hydrothermally altered equivalents [19,41]. Though considered to be of low solubility and relatively chemically inert, dissolution of a small fraction of chromite can release elevated levels of Cr.

There have been reports of elevated levels of Cr in groundwater systems worldwide, most at levels exceeding the 50 μg/L maximum contaminant level (MCL) set by the World Health Organization (WHO). Concentrations up to 700 μg/L have been reported in soil solutions in phosphate-fertilized soils in Greece, but geogenic Cr levels around the world are usually at least an order of magnitude below this value [56,58]. In the US, high concentrations of total Cr, mostly occurring as Cr(VI) have been detected at levels up to 60 μg/L in the Mojave Desert [59]. A combination of pH as high as 9 and dissolved O_2_ > 1 mg/L and fluctuating redox conditions dictated Cr speciation in groundwater sourced from alluvial aquifer materials weathered from mafic rocks in such areas [59].

### 3.3. Vanadium

Vanadium occurs in the Earth’s crust at an average content of around 2 mg/kg and is generally widely dispersed; concentrated mineral deposits are rare [60]. V tends to be present in large quantities in mafic igneous rocks, in lesser quantities in ultramafic rocks, and in very small quantities in felsic rocks [61]. There are more than 80 V-bearing minerals present in bedrock materials occurring as sulfides, sulfates, silicates, oxides, phosphates and vanadates [21]. Due to its affinity for organic rich sediments, V is also found in high concentrations in crude petroleum and shales [62]; an average concentration of 790 mg/kg have been reported in black shales around the world [63]. Major V deposits tend to occur as sandstone-hosted V, shale-hosted V, vanadate, or vanadiferous titanomagnetite, occurring in China, Russia and South Africa, as well as the US, Canada, and Australia [61]. Dispersion of V in the environment occurs due to its sensitivity to O_2_ and acidity, as well as its ability to readily substitute for both Fe and Al in both primary and secondary materials, such as clays, due to the size and charge of its trivalent ion in the environment [62]. Anthropogenic activities such as steel production, coal, petroleum production, rock phosphate fertilizers, superphosphate fertilizers and mining are also major anthropogenic sources of V into the environment [64].

In groundwater, the occurrence of V is highly dependent not only on potential anthropogenic sources, but also on the geology and aqueous conditions, and as such will be highly variable. For example, the average V concentration in California groundwater was found to be 5 μg/L. However, up to 70 μg/L V have been reported in groundwater in the northeastern San Joaquin Valley [5,6]. In some drinking water sources in Italy, values as high as 180 μg/L have been detected [5,65]. In general, higher concentrations in groundwater occur where the aqueous conditions are either alkaline, oxic, or both. [6]. High groundwater V concentrations have been documented in oxic as well as suboxic, near-neutral groundwater [6].

### 3.4. Challenges

Despite a sophisticated understanding of the mineralogy associated with Cr, V, and As, there are many challenges associated with understanding the sources of subsurface contamination with these elements. Although elements of interest can be broadly dispersed on aquifer rocks throughout geologic unit or region (as is often the case for As, Cr, and V), the elemental distribution or solid-phase speciation may be quite heterogeneous at a small scale, pointing to “hot spots” where elements are concentrated in the solid phase and become available for release to groundwater. It is worth noting that all Cr, V, and As, though ultimately derived from the sources above, are often associated as secondary deposition with Fe or Al (oxyhydr)oxides [19,49,62], and may be influenced by the distribution of solid oxidants and reductants, like Mn oxides and organic matter [66,67]. As a further complication, trace element release from (oxyhydr)oxides can be dominated by desorption, which is much more rapid than mineral dissolution, resulting in a larger element release rate relative to groundwater flow rate through the aquifer solids. The distribution of these elements in primary and secondary minerals may vary over short horizontal or vertical differences in spatial location. Such a spatially variable distribution results in an uncertain source region that is in the subsurface is the norm, not the exception, for geogenic contaminants. Consequently, most of the required information for developing a quantitative understanding of sources and mobilization mechanisms is initially laborious and site specific, whereas a region-wide synthesis to reduce population-scale exposure should evaluate and encompass a range of possibilities both in terms of solid-phase contaminant occurrence and the hydrogeochemical mechanisms that control contaminant levels in groundwater.

## 4. Biogeochemical Processes

Biogeochemical processes can couple with the hydrological and mineralogical factors above to result in the solubilization of geogenic As, Cr, and V. The evolution of groundwater composition (viz. Eh and pH, but also the dissolution other solutes) is driven by a complex array of transport and chemical reactions that together dictate the interactions between the major solid (e.g., Fe (oxyhydr)oxides and silicate minerals) and dissolved phases within the aquifer. This evolution also drives the solubilization and mobilization of lower abundance species, including geogenic contaminants. It is worth noting that contaminants often have contrasting redox controls on their solubility and mobility, meaning that conditions that favor mobilization of one element may favor sequestration of another.

### 4.1. Redox Controls on Speciation

**Figure 3.**
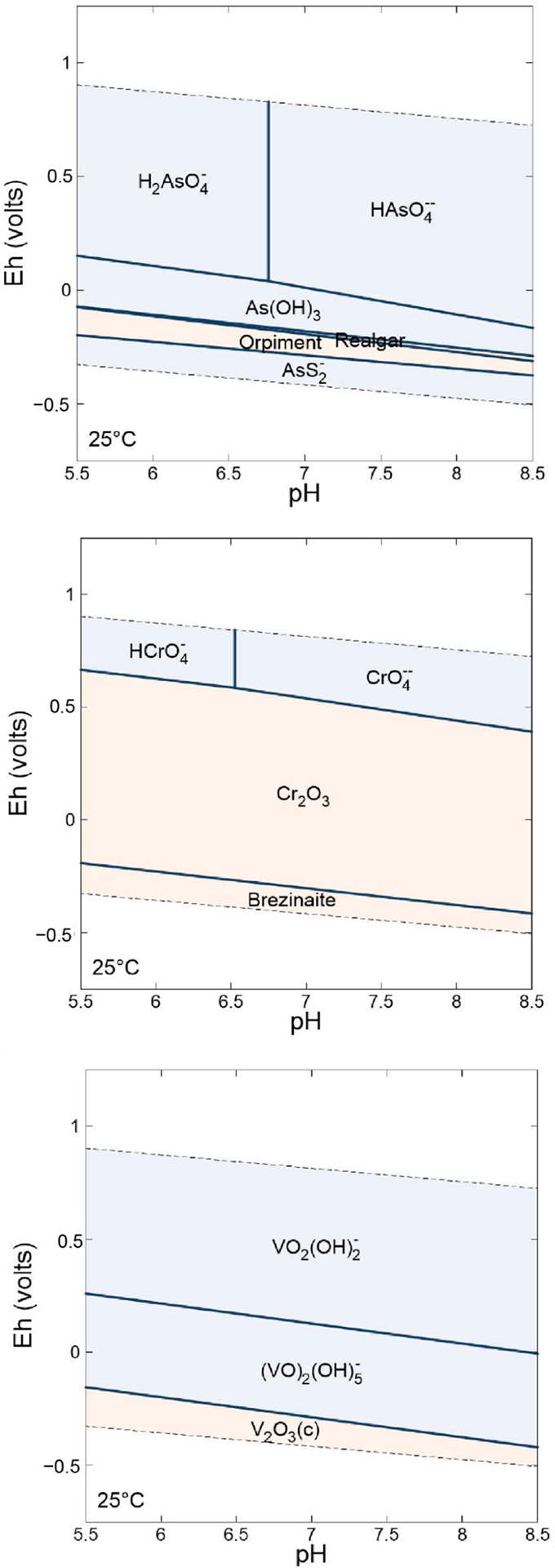
Stability diagrams for As, Cr, and V based on 10 μg/L for each element, HCO_3_^−^ and SO_4_^2−^ activities of 10^−3^ and 10^−4^ M using data from [6,69]. Figure constructed using the student version of the Geochemist’s workbench.

A predominant factor that controls the speciation therefore the solubility, mobility, and bioavailability, of As, Cr, and V is the oxidation state of the elements and pH (Figure 3). For these elements, a variety of oxidation states is possible under natural aqueous conditions (slightly acidic to slightly alkaline in most settings), and the different states result in species that can have very different solubilities. The similarities in the solubilizing conditions for Cr and V and the opposite solubilizing conditions for As help to explain the concurrences and anti-correlations of certain elements in water supplies [15,16,68].

Inorganic As, under natural conditions, is typically found as either As(III) or As(V). Pentavalent arsenic occurs predominantly as oxyanions at near-neutral to alkaline pH, whereas As(III) commonly occurs as the uncharged As(OH)_3_. In comparison to other oxyanions, including Cr and V, As retains more mobility across a wider range of groundwater pH and redox conditions, particularly under mildly reducing conditions where As(III) predominates as soluble As(OH)_3_ [70].

Chromium typically occurs in one of two oxidation states, Cr(III) and Cr(VI). In contrast to As, in which the reduced state is more soluble, Cr(VI) is known to be significantly more mobile than Cr(III), and thus Cr(VI) is more likely to be found in groundwater when conditions are oxidizing and alkaline [71].

Vanadium can occur under typical groundwater redox conditions as two different oxidation states: V(IV), dominant under low pH and suboxic conditions, and V(V), which dominates under high pH or oxic conditions. Both oxidation states can be soluble and can co-occur depending on the pH and co-occurring constituents of the groundwater [21]. However, V(IV) has been much less documented than V(V) in groundwater studies, V(IV) has been inferred based on indirect evidence [6].

### 4.2. Redox Transformations

Fluctuations in redox conditions, or the presence of other redox-active materials, can result in redox reactions that change the oxidation state of As, Cr, or V from a mobile form to an immobile form, or vice versa. The presence and concentration of redox-active elements can have an outsized effect on the redox potential of the groundwater system, which in turn affects the redox state and speciation of trace elements. Geologic or soil systems can have high concentrations of Fe and Mn [9,72], which can act as a redox buffer due to their relatively high abundance compared to other redox-sensitive elements, poising the system to a steady potential, and altering the transformations, and therefore the solubility, of trace elements such as a As, Cr or V [43,73].

**Figure 4.**
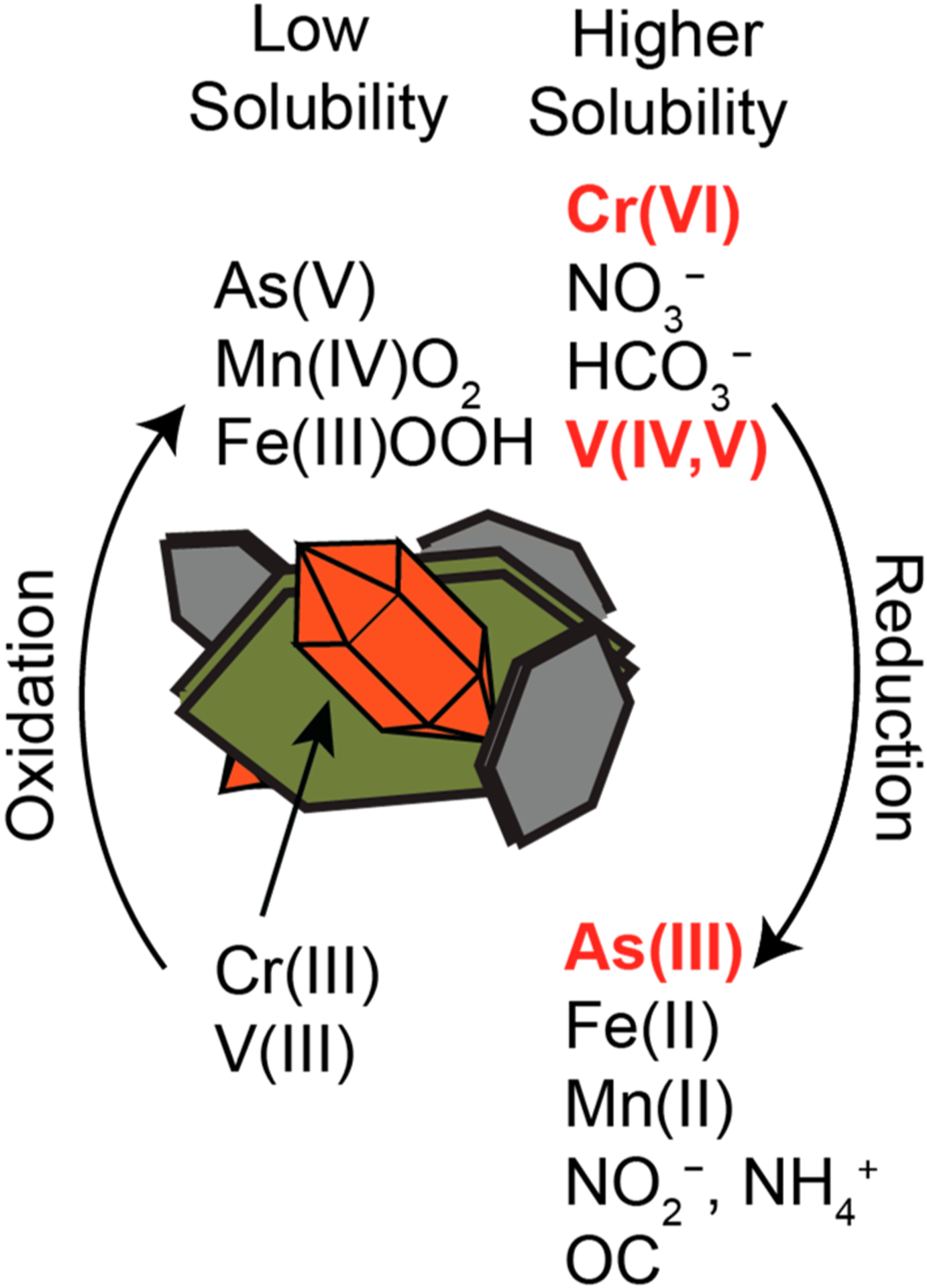
Redox controls on elemental mobility and toxicity that highlights contrasting redox controlled solubilities of As, V, and Cr. Low solubility species tend to form precipitates or sorb strongly to aquifer materials. Red indicates the oxidation states thought to pose more threat to health. OC = organic carbon.

Iron and Mn, in dissolved or solid forms, may also directly participate directly in redox reactions with Cr, V, and As (Figure 4). Manganese oxides, a primary subsurface oxidant, will oxidize As(III) to the less mobile state of As(V), a reaction that occurs more quickly, and therefore more favorably, than oxidation by Fe (oxyhydr)oxides [72]. These reactions can take seconds to days and are generally influenced by concentration of As(III), temperature, and pH. Clays can also act as reaction surfaces for the oxidation of As(III) to As(V), again, strongly influenced by pH, ionic strength, and composition of clay surfaces [49]. Likewise, Cr(III) and V(IV) can also be oxidized to Cr(VI) and V(V) by naturally occurring mineral oxidants, most commonly Mn oxides [74,75]. For Cr(III), the reaction with Mn oxides is dependent on the pH of the environment; where the pH is less than 6, the reaction occurs with dissolved Cr(III) adsorbing onto the surface of solid Mn oxides, whereas for a pH greater than 8 the reaction occurs by the redox cycling of Mn the surfaces of solid Cr(III) surfaces [74].

Reduction reactions are also significant. Cr(VI) can undergo reduction to its more insoluble Cr(III) form via redox reactions with dissolved Fe(II), Fe(II) minerals (pH > 5.5), sulfides (pH < 5.5), and organic matter [74,76]. Similar to reactions with As and Cr, Fe(II)-bearing minerals have also been suggested to actively reduce V(V) to V(III), another oxidation state that typically is only found in anoxic systems, though this reaction occurs over the course of months [77].

Although redox transformations controlling the solubility of elements can occur abiotically, microorganisms often mediate redox reactions because they utilize these transformations for energy synthesis and/or the breakdown of organic material [3,43]. As such, redox transformations tend to occur in a thermodynamically predicted sequence that corresponds to a progressively more reduced redox potential, with microbes preferentially utilizing reactions that will provide the most energy for their metabolism. This preferential order and the associated redox potential of each transformation is known as the redox ladder [78]. In organic-rich aquifers, the predominant electron donor driving microbially-mediated redox reactions is organic matter. Microbially mediated reactions can directly drive the redox environment of the groundwater system by consuming electron acceptors (O_2_, nitrate, Mn oxides, Fe (oxyhydr)oxides, and sulfate), sustaining anoxic conditions in groundwater separated from the water table, and farther along flowpaths promoting reducing conditions characterized by detectable Mn^2+^, Fe^2+^, sulfide, or methane.

Along this redox trajectory, As, Cr, and V also undergo oxidation state changes (Figure 2). In addition to interactions with abiotic species, specific organisms also oxidize or reduce As, Cr, or V, indicating that trace elements can be converted through microbially-mediated processes (in addition to overall microbially-mediated control of the redox state of groundwater or sediments). In some cases, this can lead to redox-active contaminants being utilized by microbes directly as an energy source. Vanadium, having a high reduction potential and a relatively low bacterial toxicity, has been shown to act as an electron acceptor for the respiration of a number of microbial species, including *Pseudomonas vanadium-reductans* T-1, *Pseudomonas isachenkovii* A-1, and *Geobacter metallireducens*, among others [62,79]. Arsenic-metabolizing species have also been found in both contaminated and non-contaminated sediments [79,80]. In addition to oxidation and reduction, detoxification of As via methylation is a widespread bacteria capability [80,81]. Microbial interactions with Cr tend towards detoxification mechanisms as well; Cr(VI) adsorption by the cell membrane and subsequent reduction to Cr(III) has been shown to occur in both fungi and bacteria [81].

Microbes can also influence the solubility and mobility for redox-active elements by mediating the formation of other materials in the groundwater system. Microbially created amorphous Fe and Mn (oxyhydr)oxides can directly contribute to the reactions that affect the redox potential of the groundwater system. In turn, these metal (oxyhydr)oxides can be utilized by microbes for their metabolic activities, releasing materials that are bound to the (oxyhydr)oxide surfaces, i.e., reductive dissolution. Because of widespread association of As with Fe (oxyhydr)oxides, reductive dissolution is a common mechanism of As release into groundwater, where the decay of organic matter consumes O_2_, creating anoxic conditions that promote reduction of the Fe (oxyhydr)oxide and As to the more soluble As(III) form. Similarly, V will strongly sorb to Fe (oxyhydr)oxides, and can undergo reductive dissolution in a manner analogous to As; this process can lead to an increase in both dissolved V and dissolved Fe under reduced conditions [64]. The oxidation of sulfide minerals to Fe (oxyhydr)oxides can also concomitantly release As and sulfate into the groundwater environment [13,49]. Microbes will create organic materials that can change the mobility of As, Cr, and V by acting as ligands, or by directly reducing or oxidizing the element; the redox-active element can also be released into groundwater systems when the organic materials are degraded for microbial metabolism.

### 4.3. Sorption and Desorption Reactions

In addition to redox reactions, interfacial interactions such as adsorption and desorption reactions, can affect contaminant mobility. Both As(III) and As(V) will sorb to Fe and Al (oxyhydr)oxides (particularly high surface area or poorly ordered minerals [82]), depending on the pH and competing ions. In addition to oxidizing As(III), as noted above, both Mn oxides and clays will also sorb As [49,72,83], further limiting the mobility of As. Generally, As(III) is thought to bind weakly as an outer sphere complex whereas As(V) is more likely to form strong inner sphere complexes [84]. Sorption of V is influenced also by its differently charged oxidation state. Although both the anions and cations will sorb to mineral surfaces over a wide range of pH conditions, V(IV) cations will sorb more strongly to the negatively charged (oxyhydr)oxide surfaces than the V(V) anions [5], but this is pH dependent and V(V) is known to form inner sphere complexes [84]. Adsorption and desorption to aquifer materials have been shown to be the primary control on V concentrations where groundwater conditions are oxic [6]. In contrast to As and V, the limiting factor of Cr mobility is the weathering of Cr(III) materials. Weathering enables a small amount to dissolve into the aqueous phase, where it can be adsorbed onto clay minerals or precipitated out of solution with either with (oxyhydr)oxide (Al and Fe) or with free cations (Fe(III) and Mn(II) [74,76]. In acidic environments (pH < 6), Cr(IV) oxyanions can become adsorbed by cationic colloids (usually as an inner-sphere complex [84]), an effect enhanced with increasing total organic carbon content in soil or sediment [74].

In addition to adsorption and desorption, the effect of competition between different ions can affect mobility via surfaces preferentially adsorbing one ion over another. This has been studied with As in particular, as it is ubiquitous and toxic to humans, making its sorption chemistry of particular interest. Of the common ions that appear in environmental aqueous environments, phosphate and carbonate oxyanions stand out as being competitive with As(V) for binding to the surfaces of Fe (oxyhydr)oxides [85–87]. When phosphate and carbonate are present in the groundwater system, they may be able to outcompete arsenate for inner-sphere complexation to the surface, resulting in more As in solution [85,86]. Sulfate ions also shows competitive behavior, but to a lesser extent and at least partially dependent on pH, suggesting outer-sphere complexation [85]. Coexisting major ions also modify adsorption sites, in addition to competing with As for adsorption sites. For example, sodium modifies the surface charge of As adsorption sites, resulting in higher As concentrations being found in Na-dominated groundwater rather than Ca-dominated groundwater. Na-dominated major element chemistry is common to groundwater in volcanic-source basins [4,88,89]. Dissolved organic matter, particularly humic and fulvic acids, have also been found to compete with As for mineral surface binding sites. How effectively these organic acids inhibit As adsorption has been shown to be dependent on the pH of the aqueous phase [90]. However, organic materials can also form ternary complexes with arsenate via an intermediary bridging cations, such as Fe(III), which can favor retention of As rather than release [91]. Sulfate and phosphate have also been shown to compete with Cr for adsorption sites [92], V has been shown to have differing competitive behaviors, specifically coordinating with biogenic ligand and compounds, depending on its oxidation state. Its anionic form resembles and competes with phosphate, whereas its cationic form more closely resembles the behavior of transition metal ions [93].

### 4.4. Challenges

From the sections above, it is clear that a large set of biotic and abiotic reactions control the evolution of groundwater chemistry, and thus the speciation and solubilization of As, Cr, and V. This, along with our understanding of source mineralogy and groundwater flow, allows formulation of a multiscale conceptual model (Figure 1) in which groundwater becomes contaminated with geogenic contaminants as it travels through the subsurface. In general, we have a reasonable understanding of the reactions that are important to this evolution; however, in many cases, the kinetics of these reactions are unknown or at least poorly constrained. This is made even more difficult by noting that many of the processes are: (1) strongly Eh and pH dependent; (2) involve not only oxyanionic but sometimes cationic (e.g., V(IV)) or uncharged (e.g., As(III)) species; (3) heterogenous reactions involving interactions between aquatic species, minerals, and/or solid-phase organics; (4) microbially mediated processes; and (5) kinetically controlled in the natural setting (that is, slow and may be observed at disequilibrium), which severely limits quantitative approaches to modeling of geogenic contamination of groundwater.

## 5. Measurement Methods

Critical to the study of metal(loid) distribution and fate in the natural environment is being able to determine concentration and speciation of the metal(loid) in both the aqueous-phase ground water and the solid-phase aquifer substrates. When groundwater samples are analyzed for the total concentration of metal(loid)s, there are number of standard procedures (in the US these are often proscribed by regulatory agencies) that typically involve an instrumental method using inductively coupled plasma (ICP) to vaporize and excite elements coupled to deception by optical emission spectrometry (OES) or mass spectrometry (MS). Detection by ICP-OES utilizes individual elements specific interactions with electromagnetic radiation whereas ICP-MS determines the concentration of an element in a sample by converting elements into gas phase ions and using a series of electromagnets to sort them by their mass-to-charge ratio. Selecting for the appropriate method depends on the sensitively needed, and the occurrence of analytical interferences [94]. Determining the speciation and oxidation state, of metal(loid)s in water can be conducted by coupling one of the previous analytical methods with ion chromatography, either in-line with the analytical instrument [95–97] or utilizing field-based separation techniques [98]. It should be noted that for some species (viz. Cr(VI)), specific approaches may be required to obtain adequate sensitivity quantitation in groundwater [99].

Total concentrations of metal(loid)s in aquifer solids can be achieved in several ways, depending on the elements of interest and their abundances. X-ray Fluorescence (XRF) spectroscopy utilizes high-energy X-rays to excite electrons in atoms at the surface of a solid-phase sample, and emitted fluorescence radiation is used to determine the concentrations elements in the sample. XRF has the benefit of being non-destructive and a range of instruments are available, from hand-held field instruments to more precise and sensitive laboratory-based systems; however, XRF may be limited by penetration depth of the incident X-rays, lack of ability to quantify elements with low atomic weights, and challenges in building calibration curves for quantifying elemental concentrations in complex, natural materials. Other advanced, non-destructive analytical techniques for measuring total concentrations of elements in solids, such as neutron activation analysis [100,101], are effective but often have limited availability for routine analysis of environmental samples. In contrast, total concentrations may also be assessed following total dissolution of the solid-phase material, often utilizing concentrated acids, heat and high pressures [101–103]; once dissolved, resulting aqueous samples can be analyzed by ICP-OES/MS and concentrations of elements of interest in aquifer solids may be back-calculated. Such destructive dissolution techniques have advantages of high throughputs but may be limited by the risks associated with strong acids (particularly HF) and the frequent inability to completely dissolve the solid-phase sample [104].

Approaches to understanding element-specific metal(loid) speciation in geologic materials are limited, with sequential extractions, spectroscopic methods, or microscale chemical imaging typically employed. Sequential extractions typically involve removing a target analyte (such as As, Cr, or V) using a series of extractant solutions that target increasing recalcitrant pools, often including cation-exchange extractants, carbonate-dissolving extractant, and strong acid extractants that target poorly order and crystalline minerals [105]. However, it is well established that these extracts do not exclusively solubilize materials from their target pool [106,107]. Nonetheless, this approach allows for quantitative assessment of how much of a specific element is associated with the operationally defined extractions, providing an insight on the relative availability [108]. In contrast, X-ray spectroscopic methods (commonly conducted at synchrotrons) utilize a monochromatic X-ray, probing all atoms a single element in a sample. The resulting spectra consist of two regions: an ‘edge’ region, where the adsorption of the X-rays increase rapidly and is characteristic of the element of analysis, and an extended region, characterized by longer oscillations following the edge. Analysis conducted in the edge region is known as X-ray adsorption near-edge structure spectroscopy (XANES), and is commonly used to determine the valence, and sometime fingerprint the local coordination environment, of an element. In the extended region, extended X-ray absorption fine structure spectroscopy (EXAFS), which is suitable for more concreted elements, is used to determine local coordination environment around an element [109]. Finally, mapping elemental associations and local speciation of elements in solid phases can be accomplished by a wide array of laboratory instrumentation; commonly used approaches include electron microscopy with energy dispersive spectroscopy (SEM-EDS), electron probe micro analysis (EPMA), micro X-ray fluorescence spectroscopy (μ-XRF) and secondary ion mass spectrometry (SIMS). Each of these approaches allows for spatially resolving the distributions of metal(loid)s in relation to other elements (and by association, mineral phases) of interest; however, synchrotron-based μ-XRF elemental mapping has the added benefit that specific metal(loid) oxidation states may be discerned when images are generated across a range of incident X-ray energies [110].

## 6. Mechanistic Modeling

Mechanistic modeling is the gold standard for describing environmental systems because it integrates understanding of fundamental processes into a mathematical representation of how the subject system behaves [111–117]. Such approaches have the promise of improving fundamental understanding of system behavior, predicting unobserved spatial or temporal states, and supporting design, management, and policy approaches to meet a set of constrained objectives. However, formation of incisive models, particularly those rooted in first principles, is complicated even for relatively simple systems. The development of such models in the subsurface is at the forefront of the state of science. In this section, we discuss the steps to formulate mechanistic models seeking to build on conceptual models that incorporate hydrogeochemical heterogeneity, mineralogical diversity, and biogeochemical complexity.

### 6.1. Fundamental Concepts

Subsurface systems have been modeled using mechanistic approaches both as individual systems and as components of coupled systems [111–114,117–123]. The well-established nature of mechanistic modeling of subsurface systems suggests a mature state of knowledge that belies the challenges associated with the mechanistic description of certain complex systems, such as those of focus herein. Because mechanistic models mathematically represent our fundamental understanding of a system, realistic models are underpinned by such a mature understanding [112–114,116,117,120]. When that mature understanding does not yet exist, limitations with the ability of mechanistic models to describe the system of concern naturally follows.

Even for cases in which fundamental understanding of the operative processes are well understood, challenges with mechanistic modeling can exist. For example, mechanistic models are systems of partial differential algebraic equations that necessarily require initial and boundary conditions, and model parameters (e.g., material properties, reaction rates). When these conditions vary in space and time in such a way that specification of the necessary model inputs are stochastic, uncertainty in the resultant model is a natural consequence [114,117,123–132]. Such uncertainty in mechanistic models of subsurface systems is universally the case, but often ignored by purely deterministic modeling approaches.

**Figure 5.**
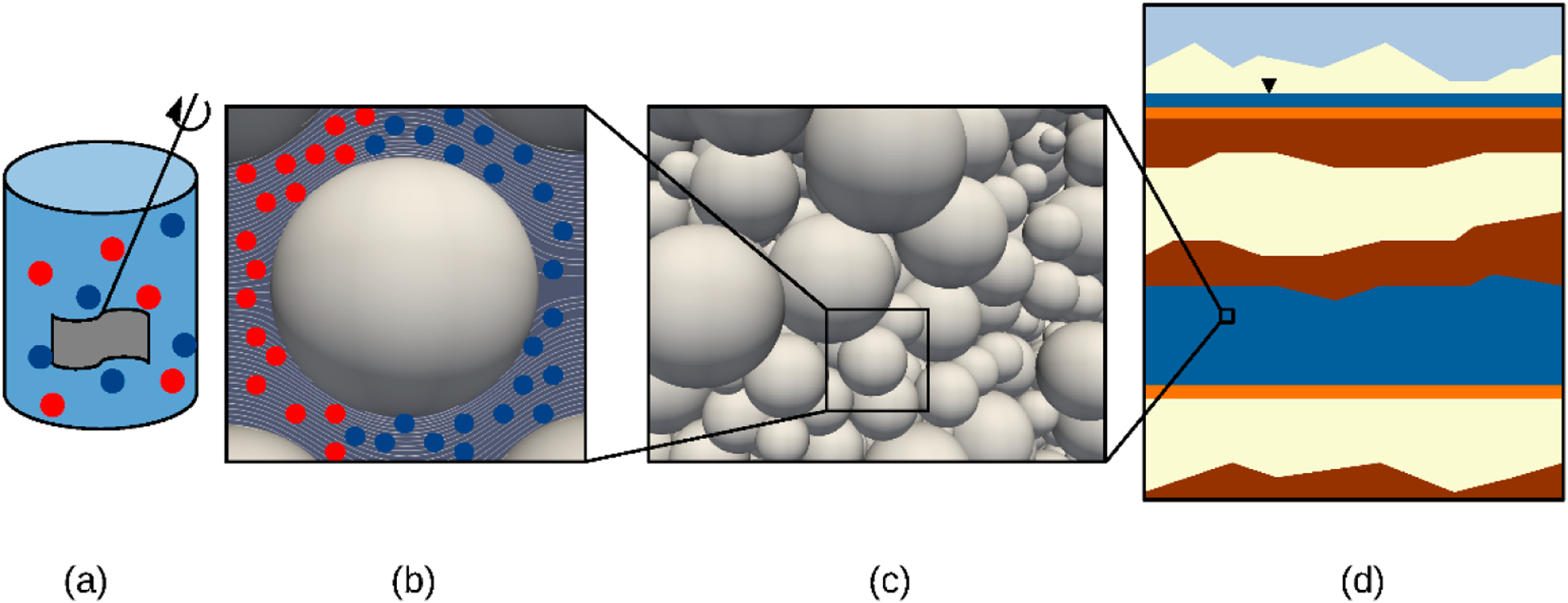
Mechanistic modeling approaches: (**a**) well-mixed batch system; (**b**) microscale modeling approach where the colored dots indicate reactive species; (**c**) a macroscale point, which can be resolved using microscale methods or averaged over at the macroscale; and (**d**) a field-scale system with a macroscale point denoted.

The issue of scale also introduces an important challenge to mechanistic modeling of certain subsurface systems. Let’s presume that at some small scale, which we will call the microscale (Figure 5), the location and properties of the solid and fluid phase distributions are fully resolved in space and time, and all processes are described accurately using mechanistic approaches. Under such conditions, a mechanistic microscale model can provide an accurate description of the system [113,117–121]. However, natural subsurface systems of concern have length scales and media complexity that preclude the use of microscale models [111–113,116,117,133,134]. To produce mechanistic representations that are tractable, a larger scale representation, called the macroscale, must be used. At the macroscale, the mechanistic representation is based upon an averaged behavior of the underlying microscale representation of the system [111–113,116,117,119,133,134]. Such a representation can be approached with varying degrees of rigor ranging from phenomenological to detailed first-principles mathematical approaches for deriving such models. Regardless of the rigor of the chosen approach, macroscale models require approximations of underlying microscale behavior that can introduce additional sources of error [114,116,117,133,135–137]. This error, referred to as modeling error, poses a challenge to macroscale modeling even if perfect knowledge of auxiliary conditions and model parameters exists.

### 6.2. Modeling Metal(loid)s

With this brief introduction into the important fundamentals of mechanistic modeling of subsurface systems, let’s consider the specific case of metal(loid)s of focus in this work. Observations of both laboratory and field-scale systems reveal the importance of geogenic sources and biogeochemical reactions as dominant factors influencing human exposure to these compounds in drinking water [83,138–142]. Mechanistic models can bridge these disparate observational scales to produce insight into system behavior that can be used to protect human health. What follows is a discussion of the contributions and challenges associated with mechanistic modeling of metal(loid)s systems that considers a range of scales that successively increase in complexity. Each successive increase in scale contributes new complexity and challenges.

### 6.3. Batch Models

Batch systems, depicted in Figure 5a, are zero dimensional in space and one-dimensional in time, making them an ideal starting point for our discussion. At the batch scale, advanced spectroscopic techniques can be used to elucidate metal speciation, mineral surface characteristics, and binding mechanisms [114,143–146]. Relatively refined descriptions of solution composition are also accessible for such systems. With such data in a well-mixed system, a mechanistic chemical species, reaction, and mass transfer model can be deduced based upon conservation of mass equations [114,141,143–146]. Mature publicly available software exists for such purposes, such as PHREEQC, TOUGHREACT, and CrunchFlow, among others [134,147,148]. Parameters and a representative model related to mass transfer to the solid phase are needed, but the homogeneous water phase reactions and corresponding rate coefficients exist in available databases [147–149]. In short, at the batch scale, mechanistic modeling of the systems of concern is relatively straightforward. The precise form of such a model will depend upon the characteristics of the solid phase and the total set of species and reactions that are considered. Such approaches lead to a large number of species and component reactions that must be considered [83,118,124,141–144,150]. In some cases, the solid particles can be further divided into a set of minerals and organic components, leading formally to a set of solid phases [114,118,141,145,146,151]. The goal of a mechanistic model is thus to describe the species-phase composition in the batch system as a function of time, and the initial conditions must be specified to render such a system solvable.

Batch models are made up of a set ordinary differential-algebraic equations with the number of equations dependent upon the number of unique non-equilibrium species-phase combinations [134,141,147]. Given the totality of subsurface and solid-phase biochemical complexity, the number of equations in such a model can number in the dozens [83,118,123,141,143,146,152]. Despite the system complexity, success in modeling the time evolution of the system and the final equilibrium state can be expected for most systems. Batch modeling is a typical starting point when modeling complicated biogeochemical systems. Agreement to batch system data is the first validation one would seek for a biogeochemical model, and such models can provide additional insight. For example, a systematic study can be used to reduce the complexity of a problem by eliminating relatively unimportant species and reactions. Such model reduction informs the model formulation and mechanistically based correlations for larger scale systems.

### 6.4. Microscale Models

Microscale models, schematically illustrated in Figure 5b, are an extension to batch models that do not require that the system is well-mixed. In addition to not being well mixed, in most microscale systems motion of the fluid phase relative to the solid phase is permissible, such as for the case of water flowing through a porous medium system. Let’s assume the common case in which the solid phase is relatively immobile compared to the fluid phase [111,118,153–155]. In this case, the motion of the fluid phase transports dissolved species through an advective process [118,148,156–158]. A model of this system would simulate fluid flow using conservation of mass and momentum equations, along with a state equation relating the fluid density to other state variables (e.g., pressure, composition, and temperature) [111–113,155,158–160]. Such a model is embodied in the celebrated Navier-Stokes equations, and readily available solvers exist to solve for a microscale velocity field for complicated fluid distributions typical of porous medium systems [154,155,160,161]. Once a velocity field is available, a set of species transport and reaction equations must be formulated and solved to model the fluid composition [118,124,141,157,162,163]. The set of species composition equations will mimic the form of the batch model and would include diffusive transport mechanisms. The key point here is that extension of the batch model to a porous medium system described at the microscale follows a well-established path that has relatively few open questions.

The firm theoretical footing of microscale models notwithstanding, challenges remain. First, the model form is challenging to solve because of the need to solve both the Navier-Stokes equations and a potentially large system of coupled, nonlinear transport and reaction equations [118,124,141,148,153,157,161,164–166]. Coupling occurs through both homogeneous phase reactions and interphase mass transfer [118,124,141,148,153,162,163,166]. If the computational challenges associated with solving a microscale model can be overcome, a model of roughly equivalent maturity and fidelity with the underlying batch model is expected—extending the mechanistic representation to porous medium systems. Mechanistic microscale modeling can be used to study the evolution of complex geochemical systems in space, for example informing the evolution of a system along a flowpath [126,155,158,163,167–170]. A limitation is that the computational burden for solving such a system will limit the domain to a size on the order of typically less than a 20 times the characteristic length of the system in each spatial dimension, and usually much less than this for readily available computational resources [148,154,155,157,171]. For an unconsolidated porous medium system, the characteristic length is often taken as the Sauter mean grain diameter [155,172–174]. Thus, practically speaking, microscale models of complex geochemical systems are restricted to very small domains compared to the length scale of natural systems of concern [129,148,153–155,157,162,167,171]. Because microscale models are based on well-accepted fundamental understanding, they can provide high-fidelity descriptions of a system that can also be used to advance macroscale models needed to represent field-scale systems [113,119,153,155,157,162–164,175].

### 6.5. Macroscale Models

The limitations on modeling at the microscale means that macroscale models are needed to simulate field-scale systems of concern, which are depicted in Figure 5c,d. The transition from microscale to macroscale models is an area of active research. Macroscale modeling of single-fluid-phase flow is well established and routinely performed in practice with abundant practical benefits well documented [111,113,148,159]. For the geochemical systems of focus in this work, a large system of transport and reaction equations must also be formulated and solved, mirroring the process of developing microscale models based upon batch systems. The increased length scale at which macroscale models are resolved means that field-scale simulations may be possible, though these come with their own computational challenges [157,159,165,176].

Formulation of an accurate macroscale representation of the complex nonlinear reactions that occur at the microscale is one of the biggest challenges facing field-scale mechanistic modeling [124,137,157]. Currently, no one macroscale approach has been found that can produce reliable simulations of complex nonlinear geochemical fate and transport in all porous medium systems of interest [124,136,137,156,157]. What has been well established is that macroscale models do not follow a similar form to their microscale counterparts, and assuming so can introduce large errors [135–137,157,177]. Mathematically, the issue arises when attempting to represent averages of nonlinear microscale quantities as a non-linear combination of macroscale averaged quantities that is of the same functional form as the microscale form [114,135,136,177]. Put another way, assuming that each region of a macroscale model can be modeled as a batch system with known inputs and outputs introduces significant errors. A parallel exists with the representation of turbulence, where non-linear velocity deviation effects have been studied for over a century without complete success [178–180]. Modeling errors introduced by macroscale models is a serious impediment to the development of useful macroscale simulators for complicated geochemical systems [124,137,157]. Additional obstacles exist when the geochemical system of concern includes both a region above and below the water table. Such two-fluid flow problems come with their own model formulation issues [153,158,159,176], although recent advances suggest a new generation of macroscale models may resolve these deficiencies [133,181–183].

Even if accurate macroscale model formulations are developed, further issues related to the establishment of accurate initial conditions for geogenic contaminant sources and complicated redox conditions would remain [126,152,169,171,184]. This would be exacerbated by long-standing issues of the identification of material parameters such as permeability and porosity that vary significantly in space but can only be observed coarsely [123,124,132,152,184]. These issues have been found to have a greater impact on species transport compared to simpler flow problems [118,124,126,129,130,152,155,171,177]. This inevitably leads to models of subsurface systems that are best represented stochastically, reflecting existing uncertainty [123,126,132,152,177,184].

### 6.6. Conceptual Illustration: Bimolecular Reactive Transport

To illustrate the modeling approaches discussed above, as well as their limitations, we will describe the case of bimolecular reactive transport, which has been extensively discussed in the literature [126,155,157,163,170,177]. The significance of this class of problem stems from the recognition that bi- and monomolecular reaction cases have been identified as the building blocks of many significant reactions of interest [170,185,186], including metal(loid)s reactions in the environment [141,187]. With this motivation, batch, microscale, and macroscale model formulations will be discussed to illustrate the general principles described in the previous sections. Modeling errors that accompany the jump from each scale to the next will also be discussed to illustrate the limitations of common modeling approaches and to highlight research opportunities.

A general bimolecular reaction may be described by the following chemical equation:

(1) A+B→AB,

where the concentration distribution of species A, B, and/or AB might be of interest in environmental systems—implying that the concentration of all species should be resolved by modeling at any scale. The first step taken to describe reactive fate and transport in a porous medium is reaction rate characterization using batch experiments. A batch system is fully mixed and may be modeled using a system of reactive conservation of mass equations given by

(2) $$\frac{\text{d}C_{iw}}{\text{d}t}-r_{iw}=0,$$

where *C_iw_* is the concentration of species *i* in the fluid phase *w*, *r_iw_* is the reaction rate, subscripts indicate quantities that are not at the macroscale, and *i* is a species index that may be A, B, or AB for the example considered here. Equation (2) is not solvable without a closure relation for *r_iw_*, which will depend upon the specific form of the reaction rate law [157,164,167]. Here, a second-order reaction rate law will be used based upon Equation (1) and written for species *AB*, given by

(3) $$r_{ABw}={\hat{k}}_{ABw}C_{Aw}C_{Bw},$$

where ${\hat{k}}_{ABw}$ is the rate constant, and *r_Aw_* = *r_Bw_* = −*r_ABw_*.

For a batch system, the concentrations of the species of interest as a function of time is the desired solution. A well-posed batch model requires that the initial concentrations of all species, *C*_*Aw*0_, *C*_*Bw*0_, and *C*_*ABw*0_ be specified. Although the model involves three species, the batch nature of the system allows for simplifications and only a single differential equation needs to be solved for this simple case, either analytically or numerically, from which the concentration of all species can be retrieved. If ${\hat{k}}_{ABw}$ is unknown, experimental observations can be used to solve an inverse problem to determine this rate parameter [157]. For many geochemical reactions, the necessary reaction rate parameters are known and tabulated in available databases [147–149].

The next level of complexity in the hierarchy of scales is the microscale. At the microscale, the concentration of dilute species is distributed in both space and time, with spatial contributions due to advective and diffusive fluxes. A common microscale model consists of a system of advective-diffusive-reactive equations (ADRE) [118,157,164,177], given by

(4) $$\frac{\partial C_{iw}}{\partial t}-{\hat{D}}_{iw}\nabla^{2}C_{iw}+v_{w}\cdot \nabla C_{iw}-r_{iw}=0,$$

where ${\hat{D}}_{iw}$ is the molecular diffusion, and *v_w_* is the velocity of the water flowing through the system. The reaction rate expression in a microscale model corresponds to the batch model for all microscale points in the domain. Additionally, the traditional ADRE relies on the assumption of Fickian diffusion at the microscale [126,157,159,161,164,177]. Although each of these assumptions could be checked, the microscale ADRE has been found to model effectively many single-fluid phase systems of interest [155,157], and would be expected to apply similarly to the fate and transport of metal(loid)s in the environment. To render the microscale model complete, *v_w_* must be known, which in general requires a solution of the Navier-Stokes equations, a continuity equation [119,152,157,161], and an equation of state for the fluid density [111–113,159,188]. Although the batch model simplified to a one equation solve, the situation is more complicated at the microscale because of the field nature of the quantities, and the need to solve for the fluid motion.

Macroscale modeling within a porous medium is ideally developed based on microscale conservation principles. Macroscale models can be formulated either by posing the model phenomenologically, such that they have a form that is similar to the corresponding microscale model, or they can be derived by direct averaging of microscale equations. Although the former approach is encountered often enough to be considered standard [114,134,137,189], the latter approach can result in models that are consistent across scales. Careful averaging from the microscale to the macroscale provides a verifiable link between scales that can allow one to check any assumptions or approximations that have been used to derive and close the model at the macroscale [113,119,157]. Although such an endeavor is technical, it has been accomplished for many systems of interest [113,115,119,122,183], including for systems experiencing reactive transport [130,190,191].

The use of phenomenological macroscale continuum models has led to well-documented modeling error [114,117,130,135,137,155,157,177,189], and some in the literature to question whether continuum models are viable for reactive transport [114,158]. To illustrate the sources of error, we will describe the commonly used macroscale ADRE, which has been phenomenologically posed, followed by a discussion of modeling error that results from this approach. The standard macroscale ADRE is given by

(5) $$\frac{\partial C^{iw}}{\partial t}-{\hat{D}}^{w}\nabla^{2}C^{iw}+v^{w}\cdot \nabla C^{iw}-r^{iw}=0,$$

where ${\hat{D}}^{w}$ is the assumed Fickian form of hydrodynamic dispersion experienced in the averaging region, and superscripts indicate macroscale quantities. Equation (5) relies on the standard assumption that the averaging region for the macroscale model is well mixed [135,136,155,159] and the porosity is constant in space and time. A result of this assumption is that the reaction rate may be expressed for species AB in our case as

(6) $$r^{ABw}={\hat{k}}_{ABw}C^{Aw}C^{Bw},$$

where ${\hat{k}}_{ABw}$ would be the same as in Equation (3). The Fickian approximation is not applicable in general for natural systems [130,155,157,161,170] and open questions exist regarding the dispersive term for reactive systems even for the limiting case where the Fickian approximation is reasonable [130,155,157,170]. These approximations lead to modeling error, and their individual contributions to error are still an open question [126,130,155,157,177]. Additionally, Equation (5) cannot be used in cases where dissolution/precipitation leads to changes to the porous medium [118,129,164,175,192], which is possible for some systems [141,187].

The phenomenological macroscale ADRE essentially amounts to treating each macroscale averaging domain as a batch system with known inputs and outputs, which is not an accurate treatment in most field cases. A classic experiment that investigated the errors introduced by Equation (5) for bimolecular reactive transport is available in the literature [135]. It was found that the macroscale ADRE regularly predicted a mass production of the bimolecular product species that was 10—20% higher than what was observed. Although this level of error may be acceptable for laboratory experiments that are on the order of centimeters, this error would accumulate rapidly, becoming intractable in field-scale simulations that have length scales on the order of kilometers, which would be expected when modeling metal(loid)s fate and transport in the environment [137,148,155,170,187]. Model solutions that maintain the phenomenological framework of the ADRE have been proposed, but most are based on hysteretic models [124,136,157]. Such models are not ideal for heavy metal reactive transport modeling, where often the history of the system is not known, where the system is heterogeneous, and/or where inverse modeling is the explicit goal of the model [124,170,187]. The next generation of macroscale simulators requires a reactive transport model that only requires knowledge of the current state of the system, as well as accessible medium parameters. Vigorous work on these issues is underway in the geochemical modeling community [116,118,134,155,185,189].

Despite the prevalence of toxic metals such as As in the environment, field-scale mechanistic reactive transport models are rarely reported in the literature, and when they are reported they rarely incorporate all relevant physics. Typically, attempts to simulate As fate and transport in groundwater are based on statistical models [39,40,193,194], or they are phenomenologically based on posited macroscale extensions to pore-scale physics [193,195–197]. Some of the most common methods that are used in the field today rely on coupling reaction modeling using PHREEQC with some other macroscale solver [193,195–197]; however, these macroscale simulators often make use of the traditional ADRE, which does not apply to most reactive transport scenarios. Recent efforts have been made to more rigorously develop mechanistic field-scale models for As adsorption by averaging up from the pore-scale [198], but the resultant models cannot be considered mature.

### 6.7. Challenges

Barriers to the development of reliable mechanistic models include theoretical issues related to upscaling nonlinear reactive transport models from the microscale to the macroscale, managing the complexity associated with biogeochemical systems that may include dozens of species and hundreds of reactions, and the stochastic aspects of subsurface systems, including source conditions for Ar, Cr, and V that can be in a variety of mineral forms that vary markedly in space in a manner that cannot be identified deterministically. Despite the significant challenges, work toward mechanistic modeling should continue to demonstrate the maturity of our level of understanding and to make inroads toward reliable predictive modeling of these complex systems. Potential paths forward include considering well-characterized model systems, complexity analysis to determine and represent the leading-order biogeochemical reaction effects as a starting point, and theoretical advancements to reduce modeling error for nonlinear transport and reaction problems in general.

## 7. Outlook and Conclusions

The release of geogenic contaminants to groundwater is controlled by the chemistry of the subsurface environment, which is in turn controlled by the hydrology and composition of the aquifer substrates. Interactions between these two result in a wide variety of potential recation involving the contaminant metal(oid)s, including: acid/base and redox transformation, solubilization, precipitation, sorption and desorption, among others. Here, we explored the common and contrasting release and retention mechanisms for As, Cr, and V. As is known to be more soluble and toxic in its reduced form, As(III), rather than its oxidized form, As(V); conversely, both Cr and V are more mobile and toxic in their oxidized forms, Cr(VI) and V(V), rather than their reduced forms, Cr(III) and V(III). The contrasting geochemistries illustrate how challenging it can be to account for all the factors that may contribute to their release, particularly in environments with multiple potential contaminants and groundwater conditions (i.e., pH, redox potential, etc.) that can alter over both distance and time. However, in many cases interactions with mineral surfaces, such as sorption, dissolution, and oxidation, have commonality among the key drivers of the processes (e.g., Fe and Mn minerals). Modeling these interactions is important for determine how the potential hazardous metal(oid)s mobilize and move in the subsurface environment, which requires connecting the hydrology, geology, and geochemistry at scales ranging from the microscale to the macroscale.

The understanding of the hydrogeochemistry, and thus efforts to model, the subsurface are hindered by the heterogeneous nature of the subsurface. It is difficult to specifically determine the concentrations and distributions of As, Cr, and V in the aquifer substrates, the exact flow of the groundwater, how the groundwater conditions evolve, and what biotic or abiotic interactions are occurring to mobilize or hinder contaminants. The complex nature of the subsurface in turn makes scaling mechanistic models from the microscale to the macroscale difficult. Constructing and validating such models is an ongoing area of research, with improvements being made in technique, approach, and computation power, as well as the expansion of basic kinetic and sorption data from related laboratory experiments.

Moving our understanding and modeling of subsurface metal(oid) contamination forward will likely require a cross-discipline effort in research. Being able to understand the complexity of the subsurface requires a variety of complementary expertise (hydrology, geochemistry, quantitative modeling), so designing projects that bring together members of different fields will facilitate the transfer of information. Drawing from experts across disciplines also reduces the potential for gaps in fundamental knowledge, and address the challenges to developing a comprehensive body of basic knowledge around the hydrogeochemistry and creating robust quantitative models. Furthermore, an important portion of this research will be making the information available to those outside of the scientific community who will have a need for it. This will include not only the general public who may need to be aware of potential issues with groundwater should they utilize it, but also stakeholders in adjacent fields. This may be as varied as public health officials, who would need to know what communities might be impacted by potentially hazardous contaminants, to land managers and environmental regulators, who would need to know where surficial inputs or spills might cause release of geogenic metal(oid)s in the groundwater. Above all, research into the solubilization and transport of metal(oid)s into groundwater, and potentially the drinking water of millions of people, is an urgent need, in order to remediate and prevent human exposure to potentially toxic, geogenic contaminants.

## References

[1] LaFayette GN; Knappett PSK; Li Y; Loza-Aguirre I; Polizzotto ML Geogenic sources and chemical controls on fluoride release to groundwater in the Independence Basin, Mexico. Appl. Geochem 2020, 123, 104787.

[2] Pichler T; Renshaw CE; Sültenfuß J Geogenic As and Mo groundwater contamination caused by an abundance of domestic supply wells. Appl. Geochem 2017, 77, 68–79.

[3] Borch T; Kretzschmar R; Kappler A; Van Cappellen P; Ginder-Vogel M; Voegelin A; Campbell K Biogeochemical redox processes and their impact on contaminant dynamics. Environ. Sci. Technol 2010, 44, 15–23. 20000681 10.1021/es9026248

[4] Vinson DS; McIntosh JC; Dwyer GS; Vengosh A Arsenic and other oxyanion-forming trace elements in an alluvial basin aquifer: Evaluating sources and mobilization by isotopic tracers (Sr, B, S, O, H, Ra). Appl. Geochem 2011, 26, 1364–1376.

[5] Wright MT; Belitz K Factors Controlling the Regional Distribution of Vanadium in Groundwater. Groundwater 2010, 48, 515–525. 10.1111/j.1745-6584.2009.00666.x 20100292

[6] Wright MT; Stollenwerk KG; Belitz K Assessing the solubility controls on vanadium in groundwater, northeastern San Joaquin Valley, CA. Appl. Geochem 2014, 48, 41–52.

[7] Oze C; Bird DK; Fendorf S Genesis of hexavalent chromium from natural sources in soil and groundwater. Proc. Natl. Acad. Sci. USA 2007, 104, 6544–6549. 17420454 10.1073/pnas.0701085104 PMC1871822

[8] Hug SJ; Winkel LHE; Voegelin AA; Berg M; Johnson CA Arsenic and Other Geogenic Contaminants in Groundwater—A Global Challenge. Chimia 2020, 74, 524. 10.2533/chimia.2020.524 32778205

[9] Gillispie EC; Austin RE; Rivera NA; Bolich R; Duckworth OW; Bradley P; Amoozegar A; Hesterberg D; Polizzotto ML Soil Weathering as an Engine for Manganese Contamination of Well Water. Environ. Sci. Technol 2016, 50, 9963–9971. 27570123 10.1021/acs.est.6b01686

[10] Ying SC; Schaefer MV; Cock-Esteb A; Li J; Fendorf S Depth Stratification Leads to Distinct Zones of Manganese and Arsenic Contaminated Groundwater. Environ. Sci. Technol 2017, 51, 8926–8932. 28695739 10.1021/acs.est.7b01121

[11] Smith AH; Lingas EO; Rahman M Contamination of drinking-water by arsenic in Bangladesh: A public health emergency. Bull. World Health Organ 2000, 78, 1093–1103. 11019458 PMC2560840

[12] Ayotte JD; Medalie L; Qi SL; Backer LC; Nolan BT Estimating the High-Arsenic Domestic-Well Population in the Conterminous United States. Environ. Sci. Technol 2017, 51, 12443–12454. 29043784 10.1021/acs.est.7b02881 PMC8842838

[13] Ravenscroft P; Brammer H; Richards K Arsenic Pollution: A Global Synthesis; Wiley: New York, NY, USA, 2009.

[14] Farkhondeh T; Samarghandian S; Azimi-Nezhad M The role of arsenic in obesity and diabetes. J. Cell. Physiol 2019, 234, 12516–12529. 30667058 10.1002/jcp.28112 PMC13484235

[15] Coyte RM; McKinley KL; Jiang S; Karr J; Dwyer GS; Keyworth AJ; Davis CC; Kondash AJ; Vengosh A Occurrence and distribution of hexavalent chromium in groundwater from North Carolina, USA. Sci. Total Environ 2020, 711, 135135. 32000345 10.1016/j.scitotenv.2019.135135

[16] Coyte RM; Vengosh A Factors Controlling the Risks of Co-occurrence of the Redox-Sensitive Elements of Arsenic, Chromium, Vanadium, and Uranium in Groundwater from the Eastern United States. Environ. Sci. Technol 2020, 54, 4367–4375. 32167307 10.1021/acs.est.9b06471

[17] Smith SM National Geochemical Database—Reformatted Data from the National Uranium Resource Evaluation (NURE) Hydrogeochemical and Stream Sediment Reconnaissance (HSSR) Program. Available online: http://mrdata.usgs.gov/nure/sediment/ (accessed on 29 September 2022).

[18] Vengosh A; Coyte R; Karr J; Harkness JS; Kondash AJ; Ruhl LS; Merola RB; Dywer GS Origin of Hexavalent Chromium in Drinking Water Wells from the Piedmont Aquifers of North Carolina. Environ. Sci. Technol. Lett 2016, 3, 409–414.

[19] Oze C; Fendorf S; Bird DK; Coleman RG Chromium geochemistry in serpentinized ultramafic rocks and serpentine soils from the Franciscan complex of California. Am. J. Sci 2004, 304, 67–101.

[20] Ścibior A; Pietrzyk Ł; Plewa Z; Skiba A Vanadium: Risks and possible benefits in the light of a comprehensive overview of its pharmacotoxicological mechanisms and multi-applications with a summary of further research trends. J. Trace Elem. Med. Biol 2020, 61, 126508. 32305626 10.1016/j.jtemb.2020.126508 PMC7152879

[21] Gustafsson JP Vanadium geochemistry in the biogeosphere—Speciation, solid-solution interactions, and ecotoxicity. Appl. Geochem 2019, 102, 1–25.

[22] Watt JAJ; Burke IT; Edwards RA; Malcolm HM; Mayes WM; Olszewska JP; Pan G; Graham MC; Heal KV; Rose NL; Vanadium: A Re-Emerging Environmental Hazard. Environ. Sci. Technol 2018, 52, 11973–11974. 30358993 10.1021/acs.est.8b05560

[23] Bundschuh J; Niazi NK; Alam MA; Berg M; Herath I; Tomaszewska B; Maity JP; Ok YS Global arsenic dilemma and sustainability. J. Hazard. Mater 2022, 436, 129197. 35739727 10.1016/j.jhazmat.2022.129197

[24] Shaji E; Santosh M; Sarath KV; Prakash P; Deepchand V; Divya BV Arsenic contamination of groundwater: A global synopsis with focus on the Indian Peninsula. Geosci. Front 2021, 12, 101079.

[25] Huang L; Wu H; van der Kuijp TJ The health effects of exposure to arsenic-contaminated drinking water: A review by global geographical distribution. Int. J. Environ. Health Res 2015, 25, 432–452. 25365079 10.1080/09603123.2014.958139

[26] Fitts CR Groundwater Science, 2nd ed.; Academic Press: Cambridge, MA, USA, 2013.

[27] Daniel CC; Dahlen PR Preliminary Hydrogeologic Assessment and Study Plan for a Regional Ground-Water Resource Investigation of the Blue Ridge and Piedmont Provinces of North Carolina; Water-Resources Investigations Report 2002–4105; US Department of the Interior, US Geological Survey: Reston, VA, USA, 2002.

[28] McMahon PB; Chapelle FH Redox Processes and Water Quality of Selected Principal Aquifer Systems. Groundwater 2008, 46, 259–271. 10.1111/j.1745-6584.2007.00385.x 18307432

[29] Lindsey B; Zimmerman T; Chapman M; Cravotta C; Szabo Z Water Quality in the Principal Aquifers of the Piedmont, Blue Ridge, and Valley and Ridge Regions, Eastern United States, 1993–2009; US Geological Survey: Reston, VA, USA, 2015.

[30] Chapelle FH Ground-Water Microbiology and Geochemistry, 2nd ed.; Wiley: New York, NY, USA, 2000.

[31] Clark I; Fritz PF Environmental Isotopes in Hydrology; Lewis Publishers: Lewis, UK, 1998.

[32] Vinson DS; Vengosh A; Hirschfeld D; Dwyer GS Relationships between radium and radon occurrence and hydrochemistry in fresh groundwater from fractured crystalline rocks, North Carolina (USA). Chem. Geol 2009, 260, 159–171.

[33] Wanty RB; Goldhaber MB Thermodynamics and kinetics of reactions involving vanadium in natural systems: Accumulation of vanadium in sedimentary rocks. Geochim. Cosmochim. Acta 1992, 56, 1471–1483.

[34] Stumm W; Morgan JJ Aquatic Chemistry, 3rd ed.; Wiley: New York, NY, USA, 1996.

[35] Lu P; Zhu C Arsenic Eh–pH diagrams at 25 °C and 1 bar. Environ. Earth Sci 2011, 62, 1673–1683.

[36] Eby GN Principles of Environmental Geochemistry; Waveland Press: Long Grove, IL, USA, 2004.

[37] Pallud C; Masue-Slowey Y; Fendorf S Aggregate-scale spatial heterogeneity in reductive transformation of ferrihydrite resulting from coupled biogeochemical and physical processes. Geochim. Cosmochim. Acta 2010, 74, 2811–2825.

[38] Wilmoth JL Redox Heterogeneity Entangles Soil and Climate Interactions. Sustainability 2021, 13, 10084.

[39] Kim D; Miranda ML; Tootoo J; Bradley P; Gelfand AE Spatial modeling for groundwater arsenic levels in North Carolina. Environ. Sci. Technol 2011, 45, 4824–4831. 21528844 10.1021/es103336s PMC3855354

[40] Connolly CT; Stahl MO; DeYoung BA; Bostick BC Surface Flooding as a Key Driver of Groundwater Arsenic Contamination in Southeast Asia. Environ. Sci. Technol 2022, 56, 928–937. 34951307 10.1021/acs.est.1c05955 PMC8766940

[41] Kazakis N; Kantiranis N; Voudouris KS; Mitrakas M; Kaprara E; Pavlou A Geogenic Cr oxidation on the surface of mafic minerals and the hydrogeological conditions influencing hexavalent chromium concentrations in groundwater. Sci. Total Environ 2015, 514, 224–238. 25666283 10.1016/j.scitotenv.2015.01.080

[42] McMahon PB; Belitz K; Reddy JE; Johnson TD Elevated Manganese Concentrations in United States Groundwater, Role of Land Surface–Soil–Aquifer Connections. Environ. Sci. Technol 2019, 53, 29–38. 30540454 10.1021/acs.est.8b04055

[43] McMahon PB; Chapelle FH; Bradley PM Evolution of Redox Processes in Groundwater. In Aquatic Redox Chemistry; American Chemical Society: Washington, DA, USA, 2011; Volume 1071, pp. 581–597.

[44] Zhang Z; Furman A Soil redox dynamics under dynamic hydrologic regimes—A review. Sci. Total Environ 2021, 763, 143026. 33143917 10.1016/j.scitotenv.2020.143026

[45] Riedel T; Kübeck C; Quirin M Legacy nitrate and trace metal (Mn, Ni, As, Cd, U) pollution in anaerobic groundwater: Quantifying potential health risk from “the other nitrate problem”. Appl. Geochem 2022, 139, 105254.

[46] FAO and UNEP. Global Assessment of Soil Pollution: Report; FAO and UNEP: Rome, Italy, 2021.

[47] Zhu C; Burden DS Mineralogical compositions of aquifer matrix as necessary initial conditions in reactive contaminant transport models. J. Contam. Hydrol 2001, 51, 145–161. 11588823 10.1016/s0169-7722(01)00132-2

[48] Nickson R; McArthur J; Burgess W; Ahmed KM; Ravenscroft P; Rahmanñ M Arsenic poisoning of Bangladesh groundwater. Nature 1998, 395, 338. 9759723 10.1038/26387

[49] Herath I; Vithanage M; Bundschuh J; Maity JP; Bhattacharya P Natural Arsenic in Global Groundwaters: Distribution and Geochemical Triggers for Mobilization. Curr. Pollut. Rep 2016, 2, 68–89.

[50] Majzlan J; Drahota P; Filippi M Parageneses and Crystal Chemistry of Arsenic Minerals. Rev. Mineral. Geochem 2014, 79, 17–184.

[51] Leonardi G; Gnagnarella P; Fletcher T Intake of inorganic arsenic from food in Hungary, Romania and Slovakia. In Arsenic Research and Global Sustainability, Proceedings of the Sixth International Congress on Arsenic in the Environment (As2016), Stockholm, Sweden, 19–23 June 2016; CRC Press: Boca Raton, FL, USA, 2016; pp. 316–317.

[52] Smedley PL; Kinniburgh DG A review of the source, behaviour and distribution of arsenic in natural waters. Appl. Geochem 2002, 17, 517–568.

[53] Morales-Simfors N; Bundschuh J; Herath I; Inguaggiato C; Caselli AT; Tapia J; Choquehuayta FEA; Armienta MA; Ormachea M; Joseph E; Arsenic in Latin America: A critical overview on the geochemistry of arsenic originating from geothermal features and volcanic emissions for solving its environmental consequences. Sci. Total Environ 2020, 716, 135564. 31918910 10.1016/j.scitotenv.2019.135564

[54] Welch AH; Westjohn DB; Helsel DR; Wanty RB Arsenic in Ground Water of the United States: Occurrence and Geochemistry. Groundwater 2000, 38, 589–604.

[55] Barnhart J Occurrences, uses, and properties of chromium. Regul. Toxicol. Pharmacol 1997, 26, S3–S7. 9380835 10.1006/rtph.1997.1132

[56] Chrysochoou M; Theologou E; Bompoti N; Dermatas D; Panagiotakis I Occurrence, Origin and Transformation Processes of Geogenic Chromium in Soils and Sediments. Curr. Pollut. Rep 2016, 2, 224–235.

[57] Kelepertzis E; Galanos E; Mitsis I Origin, mineral speciation and geochemical baseline mapping of Ni and Cr in agricultural topsoils of Thiva Valley (central Greece). J. Geochem. Explor 2013, 125, 56–68.

[58] Saputro S; Yoshimura K; Matsuoka S; Takehara K; Narsito; Aizawa J; Tennichi Y Speciation of dissolved chromium and the mechanisms controlling its concentration in natural water. Chem. Geol 2014, 364, 33–41.

[59] Ball JW; Izbicki JA Occurrence of hexavalent chromium in ground water in the western Mojave Desert, California. Appl. Geochem 2004, 19, 1123–1135.

[60] Yang B; He J; Zhang G; Guo J Chapter 3—Vanadium mineral resources. In Vanadium; Yang B, He J, Zhang G, Guo J, Eds.; Elsevier: Amsterdam, The Netherlands, 2021; pp. 33–58.

[61] Kelley KD; Scott C; Polyak DE; Kimball BE Vanadium; Professional Paper 1802-U; U.S. Geological Survey: Reston, VA, USA, 2017; 48p.

[62] Huang J-H; Huang F; Evans L; Glasauer S Vanadium: Global (bio)geochemistry. Chem. Geol 2015, 417, 68–89.

[63] Chermak JA; Schreiber ME Mineralogy and trace element geochemistry of gas shales in the United States: Environmental implications. Int. J. Coal Geol 2014, 126, 32–44.

[64] Shaheen SM; Alessi DS; Tack FMG; Ok YS; Kim K-H; Gustafsson JP; Sparks DL; Rinklebe J Redox chemistry of vanadium in soils and sediments: Interactions with colloidal materials, mobilization, speciation, and relevant environmental implications—A review. Adv. Colloid Interface Sci 2019, 265, 1–13. 30685738 10.1016/j.cis.2019.01.002

[65] Minelli L; Veschetti E; Giammanco S; Mancini G; Ottaviani M Vanadium in Italian waters: Monitoring and speciation of V(IV) and V(V). Microchem. J 2000, 67, 83–90.

[66] Fischel JS; Fischel MH; Sparks DL Advances in Understanding Reactivity of Manganese Oxides with Arsenic and Chromium in Environmental Systems. In Advances in the Environmental Biogeochemistry of Manganese Oxides; American Chemical Society: Washington, DC, USA, 2015; Volume 1197, pp. 1–27.

[67] Tang Y; Webb SM; Estes ER; Hansel CM Chromium(iii) oxidation by biogenic manganese oxides with varying structural ripening. Environ. Sci. Process. Impacts 2014, 16, 2127–2136. 25079661 10.1039/c4em00077c

[68] Thompson AK; Monti MM; Gribble MO Co-Occurrence of Metal Contaminants in United States Public Water Systems in 2013–2015. Int. J. Environ. Res. Public Health 2021, 18, 7884. 34360177 10.3390/ijerph18157884 PMC8345721

[69] Bethke CM Geochemical and Biogeochemical Reaction Modeling, 3rd ed.; Cambridge University Press: Cambridge, UK, 2022.

[70] Adriano DC Trace Elements in Terrestrial Environments, 2nd ed.; Springer: New York, NY, USA, 2001.

[71] Rai D; Eary LE; Zachara JM Environmental chemistry of chromium. Sci. Total Environ 1989, 86, 15–23. 2602932 10.1016/0048-9697(89)90189-7

[72] Oscarson DW; Huang PM; Defosse C; Herbillon A Oxidative power of Mn(IV) and Fe(III) oxides with respect to As(III) in terrestrial and aquatic environments. Nature 1981, 291, 50–51.

[73] Keimowitz AR; Mailloux BJ; Wovkulich K; Harkness J; Ross JM; Chillrud SN Manganese redox buffering limits arsenic release from contaminated sediments, Union Lake, New Jersey. Appl. Geochem 2017, 77, 24–30. 29238131 10.1016/j.apgeochem.2016.10.003 PMC5726284

[74] Liang J; Huang X; Yan J; Li Y; Zhao Z; Liu Y; Ye J; Wei Y A review of the formation of Cr(VI) via Cr(III) oxidation in soils and groundwater. Sci. Total Environ 2021, 774, 145762.

[75] Abernathy MJ; Schaefer MV; Vessey CJ; Liu H; Ying SC Oxidation of V(IV) by Birnessite: Kinetics and Surface Complexation. Environ. Sci. Technol 2021, 55, 11703–11712. 34488349 10.1021/acs.est.1c02464 PMC11938697

[76] Kotaś J; Stasicka Z Chromium occurrence in the environment and methods of its speciation. Environ. Pollut 2000, 107, 263–283. 15092973 10.1016/s0269-7491(99)00168-2

[77] O’Loughlin EJ; Boyanov MI; Kemner KM Reduction of Vanadium(V) by Iron(II)-Bearing Minerals. Minerals 2021, 11, 316.

[78] Grundl TJ; Haderlein S; Nurmi JT; Tratnyek PG Introduction to Aquatic Redox Chemistry. In Aquatic Redox Chemistry; American Chemical Society: Washington, DA, USA, 2011; Volume 1071, pp. 1–14.

[79] Masuda H Arsenic cycling in the Earth’s crust and hydrosphere: Interaction between naturally occurring arsenic and human activities. Prog. Earth Planet. Sci 2018, 5, 68.

[80] Kruger MC; Bertin PN; Heipieper HJ; Arsène-Ploetze F Bacterial metabolism of environmental arsenic—Mechanisms and biotechnological applications. Appl. Microbiol. Biotechnol 2013, 97, 3827–3841. 23546422 10.1007/s00253-013-4838-5

[81] Gutiérrez-Corona JF; Romo-Rodríguez P; Santos-Escobar F; Espino-Saldaña AE; Hernández-Escoto H Microbial interactions with chromium: Basic biological processes and applications in environmental biotechnology. World J. Microbiol. Biotechnol 2016, 32, 191. 27718146 10.1007/s11274-016-2150-0

[82] Sowers TD; Harrington JM; Polizzotto ML; Duckworth OW Sorption of arsenic to biogenic iron (oxyhydr)oxides produced in circumneutral environments. Geochim. Cosmochim. Acta 2017, 198, 194–207.

[83] Ying SC; Kocar BD; Fendorf S Oxidation and competitive retention of arsenic between iron- and manganese oxides. Geochim. Cosmochim. Acta 2012, 96, 294–303.

[84] Strawn DG; Bohn HL; O’Connor GA Soil Chemistry; John Wiley & Sons, Incorporated: Somerset, UK, 2015.

[85] Frau F; Biddau R; Fanfani L Effect of major anions on arsenate desorption from ferrihydrite-bearing natural samples. Appl. Geochem 2008, 23, 1451–1466.

[86] Neupane G; Donahoe RJ; Arai Y Kinetics of competitive adsorption/desorption of arsenate and phosphate at the ferrihydrite–water interface. Chem. Geol 2014, 368, 31–38.

[87] Marzi M; Towfighi H; Shahbazi K; Farahbakhsh M; Rinklebe J; Lima EC Adsorption and desorption characteristics of arsenic in calcareous soils as a function of time; equilibrium and thermodynamic study. Environ. Sci. Pollut. Res 2022. 10.1007/s11356-022-22310-7 35915307

[88] Currell M; Cartwright I; Raveggi M; Han D Controls on elevated fluoride and arsenic concentrations in groundwater from the Yuncheng Basin, China. Appl. Geochem 2011, 26, 540–552.

[89] Scanlon BR; Nicot JP; Reedy RC; Kurtzman D; Mukherjee A; Nordstrom DK Elevated naturally occurring arsenic in a semiarid oxidizing system, Southern High Plains aquifer, Texas, USA. Appl. Geochem 2009, 24, 2061–2071.

[90] Wang S; Mulligan CN Effect of natural organic matter on arsenic release from soilsand sediments into groundwater. Environ. Geochem. Health 2006, 28, 197–214. 16607568 10.1007/s10653-005-9032-y

[91] Cui J; Jing C A review of arsenic interfacial geochemistry in groundwater and the role of organic matter. Ecotoxicol. Environ. Saf 2019, 183, 109550. 31419698 10.1016/j.ecoenv.2019.109550

[92] Kent DB; Davis JA; Anderson LCD; Rea BA Transport of Chromium and Selenium in a Pristine Sand and Gravel Aquifer: Role of Adsorption Processes. Water Resour. Res 1995, 31, 1041–1050.

[93] Pyrzyńska K; Wierzbicki T Determination of vanadium species in environmental samples. Talanta 2004, 64, 823–829. 18969676 10.1016/j.talanta.2004.05.007

[94] Martínez-Bravo Y; Roig-Navarro AF; López FJ; Hernández F Multielemental determination of arsenic, selenium and chromium(VI) species in water by high-performance liquid chromatography-inductively coupled plasma mass spectrometry. J. Chromatogr. A 2001, 926, 265–274. 11556332 10.1016/s0021-9673(01)01062-7

[95] Sarzanini C; Bruzzoniti MC Metal species determination by ion chromatography. TrAC Trends Anal. Chem 2001, 20, 304–310.

[96] Chen Z; Mahmudur Rahman M; Naidu R Speciation of vanadium by anion-exchange chromatography with inductively coupled plasma mass spectrometry and confirmation of vanadium complex formation using electrospray mass spectrometry. J. Anal. At. Spectrom 2007, 22, 811–816.

[97] Kilibarda N; Afton SE; Harrington JM; Yan F; Levine KE Rapid speciation and determination of vanadium compounds using ion-pair reversed-phase ultra-high-performance liquid chromatography inductively coupled plasma-sector field mass spectrometry. J. Chromatogr. A 2013, 1304, 121–126. 23871564 10.1016/j.chroma.2013.06.074

[98] Roberts LC; Hug SJ; Dittmar J; Voegelin A; Saha GC; Ali MA; Badruzzaman ABM; Kretzschmar R Spatial Distribution and Temporal Variability of Arsenic in Irrigated Rice Fields in Bangladesh. 1. Irrigation Water. Environ. Sci. Technol 2007, 41, 5960–5966. 17937267 10.1021/es070298u

[99] Wendelken S; Smith G; Munch D; Zaffiro A; Zimmerman M Method 218.7: Determination of Hexavalent Chromium in Drinking Water by Ion Chromatography with Post-Column Derivatization and UV–Visible Spectroscopic Detection; Version 1.0; U.S. EPA Office of Water: Washington, DC, USA, 2011.

[100] Laul JC Neutron activation analysis of geological materials. At. Energy Rev 1979, 17, 603–695.

[101] Koeberl C Instrumental neutron activation analysis of geochemical and cosmochemical samples: A fast and reliable method for small sample analysis. J. Radioanal. Nucl. Chem 1993, 168, 47–60.

[102] Chen M; Ma LQ Comparison of Four USEPA Digestion Methods for Trace Metal Analysis Using Certified and Florida Soils. J. Environ. Qual 1998, 27, 1294–1300.

[103] Agazzi A; Pirola C Fundamentals, methods and future trends of environmental microwave sample preparation. Microchem. J 2000, 67, 337–341.

[104] Santelli RE; Cassella RJ; Arruda MAZ; Nóbrega JA Modern Strategies for Environmental Sample Preparation and Analysis. In Environmental Geochemistry in Tropical and Subtropical Environments; Drude de Lacerda L, Santelli RE, Duursma EK, Abrão JJ, Eds.; Springer: Berlin Heidelberg, Germany, 2004; pp. 37–68.

[105] Shan X; Chen B Evaluation of sequential extraction for speciation of trace metals in model soil containing natural minerals and humic acid. Anal. Chem 1993, 65, 802–807.

[106] Gleyzes C; Tellier S; Astruc M Fractionation studies of trace elements in contaminated soils and sediments: A review of sequential extraction procedures. TrAC Trends Anal. Chem 2002, 21, 451–467.

[107] Scheinost AC; Kretzschmar R; Pfister S; Roberts DR Combining Selective Sequential Extractions, X-ray Absorption Spectroscopy, and Principal Component Analysis for Quantitative Zinc Speciation in Soil. Environ. Sci. Technol 2002, 36, 5021–5028. 12523415 10.1021/es025669f

[108] Cui Y; Weng L Interpretation of heavy metal speciation in sequential extraction using geochemical modelling. Environ. Chem 2015, 12, 163–173.

[109] Kelly SD; Hesterberg D; Ravel B Analysis of Soils and Minerals Using X-ray Absorption Spectroscopy. In Methods of Soil Analysis; Ulery AL, Drees LR, Eds.; Soil Science Society of America: Madison, WI, USA, 2008.

[110] Ellison E; Mayhew L; Miller H; Templeton A Quantitative microscale Fe redox imaging by multiple energy X-ray fluorescence mapping at the Fe K pre-edge peak. Am. Mineral 2020, 105, 1812–1829.

[111] Bear J Dynamics of Fluids in Porous Media; Elsevier: New York, NY, USA, 1972.

[112] De Marsily G Quantitative Hydrogeology: Groundwater Hydrology for Engineers; Academic Press: San Diego, CA, USA, 1986.

[113] Gray WG; Miller CT Introduction to the Thermodynamically Constrained Averaging Theory for Porous Medium Systems; Springer: Cham, Switzerland, 2014.

[114] Berkowitz B; Dror I; Hansen SK; Scher H Measurements and models of reactive transport in geological media. Rev. Geophys 2016, 54, 930–986.

[115] Miller CT; Valdés-Parada FJ; Wood BD A Pedagogical Approach to the Thermodynamically Constrained Averaging Theory. Transp. Porous Media 2017, 119, 585–609.

[116] Boso F; Battiato I Homogenizability conditions for multicomponent reactive transport. Adv. Water Resour 2013, 62, 254–265.

[117] Molins S; Trebotich D; Steefel CI; Shen C An investigation of the effect of pore scale flow on average geochemical reaction rates using direct numerical simulation. Water Resour. Res 2012, 48, W03527.

[118] Liu M; Shabaninejad M; Mostaghimi P Impact of mineralogical heterogeneity on reactive transport modelling. Comput. Geosci 2017, 104, 12–19.

[119] Bowers CA; Miller CT Generalized Newtonian fluid flow in porous media. Phys. Rev. Fluids 2021, 6, 123302. 10.1103/physrevfluids.6.123302 PMC9808824 36601019

[120] Hauswirth SC; Bowers CA; Fowler CP; Schultz PB; Hauswirth AD; Weigand T; Miller CT Modeling cross model non-Newtonian fluid flow in porous media. J. Contam. Hydrol 2020, 235, 103708. 32896762 10.1016/j.jconhyd.2020.103708

[121] Weigand TM; Miller CT Microscale modeling of nondilute flow and transport in porous medium systems. Phys. Rev. E 2020, 102, 033104. 33075978 10.1103/PhysRevE.102.033104

[122] Weigand TM; Schultz PB; Giffen DH; Farthing MW; Crockett A; Kelley CT; Gray WG; Miller CT Modeling Nondilute Species Transport Using the Thermodynamically Constrained Averaging Theory. Water Resour. Res 2018, 54, 6656–6682.

[123] Yekta A; Salinas P; Hajirezaie S; Amooie MA; Pain CC; Jackson MD; Jacquemyn C; Soltanian MR Reactive transport modeling in heterogeneous porous media with dynamic mesh optimization. Comput. Geosci 2021, 25, 357–372.

[124] Donado LD; Sanchez-Vila X; Dentz M; Carrera J; Bolster D Multicomponent reactive transport in multicontinuum media. Water Resour. Res 2009, 45, W11402.

[125] Tartakovsky AM; Panzeri M; Tartakovsky GD; Guadagnini A Uncertainty Quantification in Scale-Dependent Models of Flow in Porous Media. Water Resour. Res 2017, 53, 9392–9401.

[126] Edery Y; Porta GM; Guadagnini A; Scher H; Berkowitz B Characterization of Bimolecular Reactive Transport in Heterogeneous Porous Media. Transp. Porous Media 2016, 115, 291–310.

[127] Naftaly A; Dror I; Berkowitz B Measurement and modeling of engineered nanoparticle transport and aging dynamics in a reactive porous medium. Water Resour. Res 2016, 52, 5473–5491.

[128] Crevillén-García D; Leung PK; Rodchanarowan A; Shah AA Uncertainty Quantification for Flow and Transport in Highly Heterogeneous Porous Media Based on Simultaneous Stochastic Model Dimensionality Reduction. Transp. Porous Media 2019, 126, 79–95. 30872877 10.1007/s11242-018-1114-2 PMC6390710

[129] Menke HP; Andrew MG; Blunt MJ; Bijeljic B Reservoir condition imaging of reactive transport in heterogeneous carbonates using fast synchrotron tomography—Effect of initial pore structure and flow conditions. Chem. Geol 2016, 428, 15–26.

[130] Dentz M; Le Borgne T; Englert A; Bijeljic B Mixing, spreading and reaction in heterogeneous media: A brief review. J. Contam. Hydrol 2011, 120–121, 1–17. 10.1016/j.jconhyd.2010.05.002 20561710

[131] Nissan A; Berkowitz B Reactive Transport in Heterogeneous Porous Media Under Different Péclet Numbers. Water Resour. Res 2019, 55, 10119–10129.

[132] Rubin Y Applied Stochastic Hydrogeology; Oxford University Press: New York, NY, USA, 2003.

[133] Bruning K; Miller CT Toward a New Generation of Two-Fluid Flow Models Based on the Thermodynamically-Constrained Averaging Theory. Water 2019, 11, 2260.

[134] Steefel CI; Appelo CAJ; Arora B; Jacques D; Kalbacher T; Kolditz O; Lagneau V; Lichtner PC; Mayer KU; Meeussen JCL; Reactive transport codes for subsurface environmental simulation. Comput. Geosci 2015, 19, 445–478.

[135] Gramling CM; Harvey CF; Meigs LC Reactive Transport in Porous Media: A Comparison of Model Prediction with Laboratory Visualization. Environ. Sci. Technol 2002, 36, 2508–2514. 12075812 10.1021/es0157144

[136] Gurung D; Ginn TR Mixing Ratios With Age: Application to Preasymptotic One-Dimensional Equilibrium Bimolecular Reactive Transport in Porous Media. Water Resour. Res 2020, 56, e2020WR027629.

[137] Benson DA; Aquino T; Bolster D; Engdahl N; Henri CV; Fernàndez-Garcia D A comparison of Eulerian and Lagrangian transport and non-linear reaction algorithms. Adv. Water Resour 2017, 99, 15–37.

[138] Cheng H; Hu Y; Luo J; Xu B; Zhao J Geochemical processes controlling fate and transport of arsenic in acid mine drainage (AMD) and natural systems. J. Hazard. Mater 2009, 165, 13–26. 19070955 10.1016/j.jhazmat.2008.10.070

[139] Nordstrom DK Worldwide Occurrences of Arsenic in Ground Water. Science 2002, 296, 2143–2145. 12077387 10.1126/science.1072375

[140] Nordstrom DK; Blowes DW; Ptacek CJ Hydrogeochemistry and microbiology of mine drainage: An update. Appl. Geochem 2015, 57, 3–16.

[141] Palanichamy J; Schüttrumpf H; Köngeter J; Becker T; Palani S Simulation of ammonium and chromium transport in porous media using coupling scheme of a numerical algorithm and a stochastic algorithm. Water Sci. Technol 2009, 59, 1577–1584. 19403971 10.2166/wst.2009.159

[142] Sadiq M Arsenic chemistry in soils: An overview of thermodynamic predictions and field observations. Water Air Soil Pollut 1997, 93, 117–136.

[143] Peng C; Crawshaw JP; Maitland GC; Trusler JPM Kinetics of calcite dissolution in CO_2_-saturated water at temperatures between (323 and 373) K and pressures up to 13.8 MPa. Chem. Geol 2015, 403, 74–85.

[144] Xu J; Fan C; Teng HH Calcite dissolution kinetics in view of Gibbs free energy, dislocation density, and pCO_2_. Chem. Geol 2012, 322–323, 11–18.

[145] Han J; Ro H-M Interpreting competitive adsorption of arsenate and phosphate on nanosized iron (hydr)oxides: Effects of pH and surface loading. Environ. Sci. Pollut. Res 2018, 25, 28572–28582. 10.1007/s11356-018-2897-y 30091077

[146] Limousin G; Gaudet JP; Charlet L; Szenknect S; Barthès V; Krimissa M Sorption isotherms: A review on physical bases, modeling and measurement. Appl. Geochem 2007, 22, 249–275.

[147] Parkhurst DL; Appelo CAJ Description of Input and Examples for PHREEQC Version 3: A Computer Program for Speciation, Batch-reaction, One-Dimensional Transport, and Inverse Geochemical Calculations; USGS: Reston, VA, USA, 2013.

[148] Damiani LH; Kosakowski G; Glaus MA; Churakov SV A framework for reactive transport modeling using FEniCS–Reaktoro: Governing equations and benchmarking results. Comput. Geosci 2020, 24, 1071–1085.

[149] Thoenen T; Hummel W; Berner U; Curti E The PSI/Nagra Chemical Thermodynamic Database. Nuclear Energy and Safety Research Department Laboratory for Waste Management (LES); Paul Scherrer Institute: Würenlingen, Switzerland, 2014.

[150] Yetkin EF; Pişkin Ş Sensitivity of computational fluid dynamics simulations against soft errors. Computing 2021, 103, 2687–2709.

[151] Bradford SA; Torkzaban S; Shapiro A A Theoretical Analysis of Colloid Attachment and Straining in Chemically Heterogeneous Porous Media. Langmuir 2013, 29, 6944–6952. 23687981 10.1021/la4011357

[152] Atchley AL; Maxwell RM; Navarre-Sitchler AK Using streamlines to simulate stochastic reactive transport in heterogeneous aquifers: Kinetic metal release and transport in CO_2_ impacted drinking water aquifers. Adv. Water Resour 2013, 52, 93–106.

[153] Chen L; Wang M; Kang Q; Tao W Pore scale study of multiphase multicomponent reactive transport during CO_2_ dissolution trapping. Adv. Water Resour 2018, 116, 208–218.

[154] Municchi F; Di Pasquale N; Dentz M; Icardi M Heterogeneous Multi-Rate mass transfer models in OpenFOAM^®^. Comput. Phys. Commun 2021, 261, 107763.

[155] Sole-Mari G; Bolster D; Fernàndez-Garcia D A Closer Look: High-Resolution Pore-Scale Simulations of Solute Transport and Mixing through Porous Media Columns. Transp. Porous Media 2022.

[156] Boso F; Bellin A; Dumbser M Numerical simulations of solute transport in highly heterogeneous formations: A comparison of alternative numerical schemes. Adv. Water Resour 2013, 52, 178–189.

[157] Porta GM; Chaynikov S; Thovert J-F; Riva M; Guadagnini A; Adler PM Numerical investigation of pore and continuum scale formulations of bimolecular reactive transport in porous media. Adv. Water Resour 2013, 62, 243–253.

[158] Tartakovsky AM; Trask N; Pan K; Jones B; Pan W; Williams JR Smoothed particle hydrodynamics and its applications for multiphase flow and reactive transport in porous media. Comput. Geosci 2016, 20, 807–834.

[159] Sin I; Corvisier J Multiphase Multicomponent Reactive Transport and Flow Modeling. Rev. Mineral. Geochem 2019, 85, 143–195.

[160] Greenshields CJ Openfoam User Guide; OpenFOAM Foundation Ltd.: London, UK, 2019.

[161] Rolle M; Sprocati R; Masi M; Jin B; Muniruzzaman M Nernst-Planck-based Description of Transport, Coulombic Interactions, and Geochemical Reactions in Porous Media: Modeling Approach and Benchmark Experiments. Water Resour. Res 2018, 54, 3176–3195.

[162] Tian Z; Wang J Lattice Boltzmann simulation of dissolution-induced changes in permeability and porosity in 3D CO_2_ reactive transport. J. Hydrol 2018, 557, 276–290.

[163] Bandopadhyay A; Le Borgne T; Méheust Y; Dentz M Enhanced reaction kinetics and reactive mixing scale dynamics in mixing fronts under shear flow for arbitrary Damköhler numbers. Adv. Water Resour 2017, 100, 78–95.

[164] Patel RA; Perko J; Jacques D; De Schutter G; Van Breugel K; Ye G A versatile pore-scale multicomponent reactive transport approach based on lattice Boltzmann method: Application to portlandite dissolution. Phys. Chem. Earth Parts A/B/C 2014, 70–71, 127–137.

[165] Saaltink MW; Carrera J; Ayora C On the behavior of approaches to simulate reactive transport. J. Contam. Hydrol 2001, 48, 213–235. 11285932 10.1016/s0169-7722(00)00172-8

[166] Hiorth A; Jettestuen E; Cathles LM; Madland MV Precipitation, dissolution, and ion exchange processes coupled with a lattice Boltzmann advection diffusion solver. Geochim. Cosmochim. Acta 2013, 104, 99–110.

[167] Benson DA; Pankavich S; Bolster D On the separate treatment of mixing and spreading by the reactive-particle-tracking algorithm: An example of accurate upscaling of reactive Poiseuille flow. Adv. Water Resour 2019, 123, 40–53.

[168] Engdahl NB; Benson DA; Bolster D Lagrangian simulation of mixing and reactions in complex geochemical systems. Water Resour. Res 2017, 53, 3513–3522.

[169] Paster A; Bolster D; Benson DA Connecting the dots: Semi-analytical and random walk numerical solutions of the diffusion–reaction equation with stochastic initial conditions. J. Comput. Phys 2014, 263, 91–112.

[170] Sole-Mari G; Fernàndez-Garcia D; Rodríguez-Escales P; Sanchez-Vila X A KDE-Based Random Walk Method for Modeling Reactive Transport With Complex Kinetics in Porous Media. Water Resour. Res 2017, 53, 9019–9039.

[171] Engdahl NB; Aquino T Considering the utility of backward-in-time simulations of multi-component reactive transport in porous media. Adv. Water Resour 2018, 119, 17–27.

[172] Schopf SO; Hartwig A; Fritsching U; Mädler L Imbibition into Highly Porous Layers of Aggregated Particles. Transp. Porous Media 2017, 119, 119–141.

[173] Van Lopik JH; Snoeijers R; van Dooren TCGW; Raoof A; Schotting RJ The Effect of Grain Size Distribution on Nonlinear Flow Behavior in Sandy Porous Media. Transp. Porous Media 2017, 120, 37–66.

[174] Li L; Ma W Experimental Study on the Effective Particle Diameter of a Packed Bed with Non-Spherical Particles. Transp. Porous Media 2011, 89, 35–48.

[175] Kang Q; Chen L; Valocchi AJ; Viswanathan HS Pore-scale study of dissolution-induced changes in permeability and porosity of porous media. J. Hydrol 2014, 517, 1049–1055.

[176] Radu FA; Pop IS Newton method for reactive solute transport with equilibrium sorption in porous media. J. Comput. Appl. Math 2010, 234, 2118–2127.

[177] Tartakovsky AM; de Anna P; Le Borgne T; Balter A; Bolster D Effect of spatial concentration fluctuations on effective kinetics in diffusion-reaction systems. Water Resour. Res 2012, 48, W02526.

[178] Undheim O; Andersson HI; Berge E Non-Linear, Microscale Modelling of the Flow Over Askervein Hill. Bound.-Layer Meteorol 2006, 120, 477–495.

[179] Lian YP; Dallmann J; Sonin B; Roche KR; Packman AI; Liu WK; Wagner GJ Double Averaging Analysis Applied to a Large Eddy Simulation of Coupled Turbulent Overlying and Porewater Flow. Water Resour. Res 2021, 57, e2021WR029918.

[180] Hanjalić K; Launder BE Reassessment of modeling turbulence via Reynolds averaging: A review of second-moment transport strategy. Phys. Fluids 2021, 33, 091302.

[181] Gray WG; Bruning K; Miller CT Non-hysteretic functional form of capillary pressure in porous media. J. Hydraul. Res 2019, 57, 747–759.

[182] McClure JE; Armstrong RT; Berrill MA; Schlüter S; Berg S; Gray WG; Miller CT Geometric state function for two-fluid flow in porous media. Phys. Rev. Fluids 2018, 3, 084306.

[183] Miller CT; Bruning K; Talbot CL; McClure JE; Gray WG Nonhysteretic Capillary Pressure in Two-Fluid Porous Medium Systems: Definition, Evaluation, Validation, and Dynamics. Water Resour. Res 2019, 55, 6825–6849.

[184] Tartakovsky DM; Dentz M; Lichtner PC Probability density functions for advective-reactive transport with uncertain reaction rates. Water Resour. Res 2009, 45, W07414.

[185] Perez LJ; Hidalgo JJ; Dentz M Upscaling of Mixing-Limited Bimolecular Chemical Reactions in Poiseuille Flow. Water Resour. Res 2019, 55, 249–269.

[186] Gillespie DT The chemical Langevin equation. J. Chem. Phys 2000, 113, 297–306.

[187] Mayer KU; Benner SG; Blowes DW Process-based reactive transport modeling of a permeable reactive barrier for the treatment of mine drainage. J. Contam. Hydrol 2006, 85, 195–211. 16554107 10.1016/j.jconhyd.2006.02.006

[188] Hayes RE; Afacan A; Boulanger B; Shenoy AV Modelling the flow of power law fluids in a packed bed using a volumeaveraged equation of motion. Transp. Porous Media 1996, 23, 175–196.

[189] Carrera J; Saaltink MW; Soler-Sagarra J; Wang J; Valhondo C Reactive Transport: A Review of Basic Concepts with Emphasis on Biochemical Processes. Energies 2022, 15, 925.

[190] Massoudieh A; Dentz M Upscaling non-linear reactive transport in correlated velocity fields. Adv. Water Resour 2020, 143, 103680.

[191] Miller CT; Gray WG; Schrefler BA A continuum mechanical framework for modeling tumor growth and treatment in two- and three-phase systems. Arch. Appl. Mech 2022, 92, 461–489. 35811645 10.1007/s00419-021-01891-8 PMC9269988

[192] Noiriel C; Steefel CI; Yang L; Bernard D Effects of pore-scale precipitation on permeability and flow. Adv. Water Resour 2016, 95, 125–137.

[193] Jakobsen R; Kazmierczak J; Sø HU; Postma D Spatial Variability of Groundwater Arsenic Concentration as Controlled by Hydrogeology: Conceptual Analysis Using 2-D Reactive Transport Modeling. Water Resour. Res 2018, 54, 10,254–10,269. 10.1029/2018WR023685 PMC6472640 31007297

[194] Goovaerts P; AvRuskin G; Meliker J; Slotnick M; Jacquez G; Nriagu J Geostatistical modeling of the spatial variability of arsenic in groundwater of southeast Michigan. Water Resour. Res 2005, 41, W07013.

[195] Parkhurst DL; Kipp KL; Charlton SR PHAST Version 2-A Program for Simulating Groundwater Flow, Solute Transport, and Multicomponent Geochemical Reactions; Techniques and Methods 6-A35; US Geological Survey: Reston, VA, USA, 2010.

[196] Nikolaidis NP; Dobbs GM; Lackovic JA Arsenic removal by zero-valent iron: Field, laboratory and modeling studies. Water Res 2003, 37, 1417–1425. 12598205 10.1016/S0043-1354(02)00483-9

[197] Rawson J; Prommer H; Siade A; Carr J; Berg M; Davis JA; Fendorf S Numerical Modeling of Arsenic Mobility during Reductive Iron-Mineral Transformations. Environ. Sci. Technol 2016, 50, 2459–2467. 26835553 10.1021/acs.est.5b05956

[198] Pillai K; Raizada A Modeling Transport and Adsorption of Arsenic Ions in Iron-Oxide Laden Porous Media. Part I: Theoretical Developments. Water 2021, 13, 779.

